# Congenital anomaly and perinatal outcome following blastocyst‐ *vs* cleavage‐stage embryo transfer: systematic review and network meta‐analysis

**DOI:** 10.1002/uog.26019

**Published:** 2023-01-03

**Authors:** C. Siristatidis, M. Papapanou, V. Karageorgiou, W. P. Martins, I. Bellos, D. M. Teixeira, N. Vlahos

**Affiliations:** ^1^ Assisted Reproduction Unit, Second Department of Obstetrics and Gynecology, Medical School National and Kapodistrian University of Athens, “Aretaieion” University Hospital Athens Greece; ^2^ Obstetrics, Gynecology and Reproductive Medicine Working Group, Society of Junior Doctors Athens Greece; ^3^ University of Exeter Exeter UK; ^4^ SEMEAR fertilidade, Reproductive Medicine Ribeirão Preto Brazil; ^5^ Sotiria General Hospital National and Kapodistrian University of Athens Athens Greece

**Keywords:** assisted reproduction, blastocyst, cleavage, congenital anomaly, *in‐vitro* fertilization, perinatal outcome

## Abstract

**Objectives:**

To compare the reported rate of any congenital anomaly and perinatal outcome of pregnancy following blastocyst‐ *vs* cleavage‐stage embryo transfer using a pairwise meta‐analysis and to evaluate the same outcomes following fresh‐blastocyst, frozen‐blastocyst, fresh‐cleavage or frozen‐cleavage embryo transfer using a network meta‐analysis.

**Methods:**

A literature search was performed in PubMed, Scopus and CENTRAL and registers for ongoing studies, from inception to February 2022, for randomized controlled trials (RCTs) with any sample size and observational studies including at least 100 live births per group, comparing the rates of any congenital anomaly and perinatal outcome of pregnancy following fresh/frozen embryo transfer at cleavage (day 2–3) *vs* blastocyst (day 5–7) stage. Risk ratios (RRs) along with their 95% CIs were pooled via a random‐effects model meta‐analysis. Within a frequentist network meta‐analysis framework, outcomes of all four treatment modalities (i.e. fresh‐blastocyst, fresh‐cleavage, frozen‐blastocyst, frozen‐cleavage) were compared further. Any congenital anomaly constituted the primary outcome, whereas preterm delivery (delivery < 37 weeks), low birth weight (LBW; < 2500 g), gender of the neonate (male), perinatal death and healthy neonate (defined as liveborn neonate, delivered at term, weighing ≥ 2500 g, surviving for at least 28 days postbirth and without any congenital anomaly) were considered as secondary outcomes. Subgroup analyses by plurality (liveborn singleton *vs* multiple pregnancy) were conducted in the pairwise and network meta‐analyses. The risk of bias was assessed using the RoB2 tool for RCTs and the ROBINS‐I tool for non‐randomized studies. Certainty of evidence was assessed using GRADE.

**Results:**

Through the literature search, 550 studies were retrieved and 33 were included in the systematic review. We found no significant difference in the risk for any congenital anomaly between blastocyst‐ and cleavage‐stage transfer (RR, 0.80 (95% CI, 0.63–1.03); 10 studies; *n* = 192 442; *I* ^2^ = 85.5%). An increased probability of a male neonate was observed following blastocyst‐ *vs* cleavage‐stage transfer (RR, 1.07 (95% CI, 1.06–1.09); 18 studies; *n* = 227 530; *I* ^2^ = 32.7%). No significant differences in other secondary outcomes or significant subgroup differences between liveborn singletons and multiple pregnancies were observed.

The network meta‐analysis showed a significantly lower risk for LBW following frozen‐blastocyst *vs* fresh‐blastocyst (RR, 0.76 (95% CI, 0.60–0.95)) or fresh‐cleavage (RR, 0.74 (95% CI, 0.59–0.93)) transfer. Frozen‐blastocyst transfer was associated with an increased risk for perinatal death compared with the fresh‐cleavage method (RR, 2.06 (95% CI, 1.10–3.88)). The higher probability of a male neonate following blastocyst transfer remained evident in the network comparisons. All outcomes were assessed to be of very‐low certainty of evidence.

**Conclusions:**

Current very‐low certainty of evidence shows that there may be little‐to‐no difference in the risk for congenital anomaly or adverse perinatal outcome of pregnancy following blastocyst‐ *vs* cleavage‐stage embryo transfer, although there was a slightly increased probability of a male neonate following blastocyst transfer. When considering cryopreservation, frozen‐blastocyst transfer was associated with a reduction in the risk for LBW compared with both fresh‐transfer modalities, and fresh‐cleavage transfer may be associated with a reduction in the risk for perinatal death compared with frozen‐blastocyst transfer. High‐quality RCTs with separate data on fresh and frozen cycles and consistent reporting of culture conditions and freezing methods are mandatory. Individual participant data meta‐analyses are required to address the substantial inconsistency resulting from current aggregate data approaches. © 2022 The Authors. *Ultrasound in Obstetrics & Gynecology* published by John Wiley & Sons Ltd on behalf of International Society of Ultrasound in Obstetrics and Gynecology.

## Abstract 2

This article's abstract has been translated into Spanish and Chinese. Follow the links from the abstract to view the translations.

## RESUMEN

**Anomalías congénitas y resultados perinatales tras la implantación de blastocistos en comparación con la de embriones en etapa de escisión: revisión sistemática y metaanálisis en red**

**Objetivo:**

Comparar la tasa publicada de cualquier anomalía congénita y el resultado perinatal del embarazo tras la implantación de blastocistos en comparación con la de embriones en etapa de escisión mediante un metaanálisis por pares y evaluar los mismos resultados tras la implantación de embriones frescos de blastocisto, congelados de blastocisto, frescos de escisión o congelados de escisión mediante un metaanálisis en red.

**Métodos:**

Se realizó una búsqueda bibliográfica en PubMed Scopus y CENTRAL y registros de estudios en curso, desde su inicio hasta febrero de 2022, de ensayos controlados aleatorizados (ECA) con cualquier tamaño de muestra y estudios observacionales que incluyeran al menos 100 nacimientos vivos por grupo, que compararan las tasas de cualquier anomalía congénita y el resultado perinatal del embarazo tras la implantación de embriones frescos/congelados en el momento de la escisión (día 2–3) en comparación con la etapa de blastocisto (día 5–7). Los cocientes de riesgo (CR) junto con sus IC 95% se agruparon mediante un metaanálisis de modelo de efectos aleatorios. Los resultados de las cuatro modalidades de tratamiento (blastocisto fresco, escisión fresco, blastocisto congelado y escisión congelado) se compararon además mediante un metaanálisis en red frecuentista. Cualquier anomalía congénita constituyó el resultado primario, mientras que el parto prematuro (parto <37 semanas), el bajo peso al nacer (BPN; <2500 g), el sexo del recién nacido (varón), la muerte perinatal y el recién nacido sano (definido como recién nacido vivo, nacido a término, con un peso ≥2500 g, que sobrevive al menos 28 días después del parto y sin ninguna anomalía congénita) se consideraron como los resultados secundarios. Los análisis de subgrupos por pluralidad (nacidos vivos de feto único frente a embarazo múltiple) se realizaron en los metaanálisis por pares y en red. El riesgo de sesgo se evaluó mediante la herramienta RoB2 para los ECA y la herramienta ROBINS‐I para los estudios no aleatorizados. La certeza de la evidencia se evaluó mediante GRADE.

**Resultados:**

Mediante la búsqueda bibliográfica se identificaron 550 estudios y se incluyeron 33 en la revisión sistemática. No se encontraron diferencias significativas en el riesgo de cualquier anomalía congénita entre la implantación en etapa de blastocisto y en etapa de escisión (CR, 0,80 [IC 95%, 0,63–1,03]; 10 estudios; n=192 442; I^2^ =85,5%). Se observó un aumento de la probabilidad de que el recién nacido fuera varón tras el blastocisto frente a la escisión (CR, 1,07 (IC 95%, 1,06–1,09); 18 estudios; n=227 530; I^2^ =32.7%). No se observaron diferencias significativas en otros resultados secundarios ni diferencias significativas por subgrupos entre los embarazos nacidos vivos de feto único y los embarazos múltiples. El metaanálisis en red mostró un riesgo significativamente menor de BPN tras la implantación de blastocistos congelados frente a la de frescos (CR, 0,76 [IC 95%: 0,60–0,95]) o la de escisión frescos (CR, 0,74 [IC 95%: 0,59–0,93]). La implantación de blastocistos congelados se asoció a un mayor riesgo de muerte perinatal en comparación con el método de escisión en fresco (CR, 2,06 [IC 95%, 1,10–3,88]). La mayor probabilidad de un recién nacido varón tras la implantación de blastocistos siguió siendo evidente en las comparaciones en red. Se consideró que todos los resultados tuvieron una certeza de la evidencia muy baja.

**Conclusiones:**

La certeza actual tan baja de la evidencia muestra que puede haber poca o ninguna diferencia en el riesgo de anomalías congénitas o resultados perinatales adversos del embarazo tras la implantación de un blastocisto en el útero frente a la implantación de embriones en etapa de escisión, aunque hubo un ligero aumento de la probabilidad de un neonato varón tras la implantación de blastocistos. Al considerar la criopreservación, la implantación de blastocistos congelados se asoció con una reducción del riesgo de BPN en comparación con ambas modalidades de implantación en fresco, y la implantación en fresco en el momento de la escisión puede asociarse con una reducción del riesgo de muerte perinatal en comparación con la implantación de blastocistos congelados. Son obligatorios los ECA de alta calidad con datos separados sobre los ciclos en fresco y congelados así como informar habitualmente sobre las condiciones de cultivo y los métodos de congelación. Los metaanálisis de datos de participantes individuales son necesarios para abordar la heterogeneidad sustancial resultante de los planteamientos actuales de datos agregados.

## 摘要

囊胚期与卵裂期胚胎移植后的先天性异常和围产期结局 : 系统回顾和网络荟萃分析

**目的** 采用配对荟萃分析比较囊胚期与卵裂期胚胎移植后的任何先天性异常报告率和围产期结局，并采用网络荟萃分析评估新鲜囊胚、冷冻囊胚、新鲜卵裂或冷冻卵裂胚胎移植后的相同结局。

**方法** 在PubMed Scopus和CENTRAL及注册中心进行文献检索，寻找从开始到2022年2月正在进行的任何样本量的随机对照试验(RCT)和包括每组至少100例活产的观察性研究，比较新鲜/冷冻胚胎在卵裂(第2‐3天)与囊胚(第5‐7天)阶段移植后的任何先天性异常率和围产期结局。风险比(RR)及其95%置信区间通过随机效应模型荟萃分析汇集。在经常性网络荟萃分析框架内，进一步比较了所有四种处理方式(即新鲜囊胚、新鲜卵裂、冷冻囊胚、冷冻卵裂)的结果。任何先天性异常构成主要结果，而早产(37周前分娩)、低出生体重(LBW；<2500克)、新生儿性别(男性)、围产期死亡和健康新生儿(定义为活产新生儿，足月分娩，体重≥2500克，出生后至少存活28天且没有任何先天性异常)被视为次要结果。在配对和网络荟萃分析中，进行了多元(活产单胎与多胎)亚组分析。对RCT使用RoB2工具，对非随机研究使用ROBINS‐I工具评估偏倚风险。证据的确定性使用GRADE进行评估。

**结果** 通过文献检索，共检索到550项研究，其中33项被纳入系统回顾。我们发现在囊胚期和卵裂期移植之间，任何先天性异常的风险没有显著差异(RR，0.80(95%CI，0.63‐1.03)；10项研究；n=192 442；I^2^=85.5%)。囊胚期与卵裂期移植后，男性新生儿的概率增加(RR，1.07(95%CI，1.06‐1.09)；18项研究；n=227 530；I2 =32.7%)。其他次要结果没有明显差异，也没有观察到活产单胎和多胎之间的明显亚组差异。

网络荟萃分析显示，冷冻囊胚相比新鲜囊胚(RR，0.76(95%CI，0.60‐0.95))或新鲜卵裂(RR，0.74(95%CI，0.59‐0.93))移植后出现LBW的风险明显降低。与新鲜卵裂法相比，冷冻囊胚移植与围产期死亡的风险增加有关(RR，2.06(95%CI，1.10‐3.88))。在网络比较中，囊胚移植后男性新生儿的概率较高，这一点仍然很明显。经评估，所有结果的证据确定性都很低。

**结论** 目前非常低的证据确定性显示，尽管囊胚移植后男性新生儿的概率略有增加，在囊胚期与卵裂期胚胎移植后，妊娠的先天性异常或不良围产期结局的风险可能几乎没有差异。当考虑到冷冻保存时，与两种新鲜胚胎移植方式相比，冷冻囊胚移植与LBW风险的降低有关，而新鲜卵裂移植与冷冻囊胚移植相比，可能与围产期死亡风险的降低有关。必须有高质量的RCT，具备关于新鲜和冷冻周期的单独数据，以及关于培养条件和冷冻方法的一致报告。需要对单个参与者的数据进行荟萃分析，以解决目前汇总数据方法所导致的大量不一致。

**CONTRIBUTION**

*What are the novel findings of this work?*

This is the first systematic review and network meta‐analysis to compare the risk for any congenital anomaly and adverse perinatal outcome among all possible embryo transfer modalities, stratified according to developmental stage at transfer and cryopreservation (i.e. fresh‐blastocyst, fresh‐cleavage, frozen‐blastocyst and frozen‐cleavage transfers).

*What are the clinical implications of this work?*

Given the increasing prevalence of cryopreservation and extended culture of embryos, the findings of this work are important, as they provide information on the potential advantages and disadvantages of each type of embryo transfer, including the fresh‐blastocyst, fresh‐cleavage, frozen‐blastocyst and frozen‐cleavage interventions, based on currently available evidence.

## INTRODUCTION

Cleavage‐stage embryo transfer following controlled ovarian hyperstimulation for assisted reproduction used to be the most common method of embryo transfer. Extending embryo culture to the blastocyst stage (day 5–7) has been suggested to reduce the risk of multiple pregnancy without compromising the pregnancy rate^1^, ^2^, ^3^. Theoretical advantages of longer cell culture include the improved selection of higher‐quality embryos, better embryo–endometrium synchronization and reduced uterine contractility^4^, ^5^, ^6^, ^7^. However, the extended embryo culture increases the exposure of the embryo to cell culture conditions and may increase epigenetic alterations^8^. Clinical evidence also remains conflicting; while some studies report perinatal complications to occur more frequently in pregnancies following embryo transfer at the blastocyst stage^9^, ^10^, ^11^, ^12^, ^13^, others have reached different conclusions^14^, ^15^, ^16^, ^17^, ^18^. There is currently no high‐quality evidence to support the use of either strategy^2^.

Since the publication of previous systematic reviews^9^, ^19^, ^20^, ^21^, additional original studies have emerged^16^, ^18^, ^22^, ^23^, ^24^, ^25^, including those with a randomized design^26^, ^27^. Moreover, previous meta‐analyses merged data from fresh‐ and frozen‐cycle embryo transfers, thus not evaluating the effects of cryopreservation and developmental stage at the time of transfer on their results^19^, ^28^, ^29^. Other limitations included the observational design of the eligible studies^28^ and the fact that variations in assisted reproduction procedures, including culture conditions and media used, may have affected the final results^17^, ^30^. Even though the most recent systematic review investigated explicitly the potential impact of cryopreservation on perinatal outcome via subgroup analysis, it was not designed to compare all the available modalities^31^. Finally, previous reviews^9^, ^19^, ^20^, ^21^, ^31^ focused primarily on singleton pregnancy; however, large cohorts^14^, ^18^ providing data on infants from multiple pregnancy have emerged.

The objective of this systematic review and meta‐analysis was to compare the rate of any congenital anomaly and the perinatal outcome of singleton and multiple pregnancy resulting from blastocyst‐ *vs* cleavage‐stage embryo transfer. This work further aimed to compare the same outcomes between pregnancies resulting from fresh‐blastocyst, frozen‐blastocyst, fresh‐cleavage and frozen‐cleavage embryo transfers by using a frequentist network meta‐analysis framework.

## METHODS

### Protocol and registration

The protocol followed the updated Preferred Reporting Items for Systematic Reviews and Meta‐Analyses (PRISMA) guidelines^32^ and was registered with PROSPERO in July 2020 (registration number: CRD42020179040)^33^.

### Eligibility criteria

Eligible studies recruited women with a delivery following fresh‐ or frozen‐embryo transfer at the cleavage or blastocyst stage. The comparisons were made between pregnancies resulting from embryo transfers at the cleavage (day 2–3) *vs* blastocyst (day 5–7) stage. Randomized controlled trials (RCTs) with any sample size and observational studies (i.e. cohort or case–control studies) including at least 100 live births following cleavage‐stage embryo transfer and 100 live births following blastocyst‐stage embryo transfer were considered eligible.

The primary outcome was the risk of any congenital anomaly, either external or internal, minor or major, as defined by the World Health Organization (WHO)^34^ and the Centers for Disease Control and Prevention^35^. The rationale for merging the categories of major and minor congenital anomalies into one outcome (defined as any congenital anomaly) included practical reasons related to statistical power.

Secondary outcomes included: (i) gestational age at delivery (defined as birth in which at least one baby was liveborn), classified into term delivery (TD; ≥ 37 weeks of gestation), preterm delivery (PTD; 32–36 weeks) and very‐preterm delivery (VPTD; < 32 weeks); (ii) birth weight of liveborn infants, classified into very‐low birth weight (VLBW; < 1500 g), low birth weight (LBW; 1500–2499 g), normal birth weight (NBW; 2500–3999 g), high birth weight (HBW; 4000–4499 g) and very‐high birth weight (VHBW; ≥ 4500 g); (iii) gender of the neonate; (iv) healthy neonate, defined as liveborn neonate, delivered at term, weighing ≥ 2500 g, surviving for at least 28 days postbirth and not having any congenital anomaly; and (v) perinatal death, defined as death of the fetus/neonate between 28 weeks of gestation and 7 days after birth.

The published protocol regarding birth weight and gestational age at delivery outcomes was elaborated. Given the limited data availability, the risk of VLBW/LBW *vs* NBW/HBW/VHBW was compared between blastocyst and cleavage groups. LBW and VLBW were merged into a single category (i.e. LBW < 2500 g) and recorded as an event, and all other categories (NBW, HBW and VHBW) were used as the comparator. In the same way, VPTD and PTD were merged into a single‐event category (i.e. gestational age at delivery < 37 weeks or PTD).

### Literature search

The following databases/registers were screened: MEDLINE (accessed through PubMed), Scopus, CENTRAL, ClinicalTrials.gov and WHO ICTRP. No date restrictions were applied. The following search algorithm was used: “embryo* AND (cleav* OR “day 2” OR “day 3”) AND (blastoc* OR “day 5” OR “day 6”) AND (“Congenital Abnormalities”[MeSH] OR perinatal OR “birth weight*” OR “preterm birth*”)”. The search algorithm was adjusted for each database while maintaining a common overall architecture. The date of the initial search was 6 June 2021. The literature search was repeated on 6 February 2022 to prevent the potential omission of any recently published eligible studies.

Retrieved records underwent semiautomatic deduplication using EndNote 20 (Clarivate Analytics, London, UK)^36^. The title and abstract of all retrieved articles were screened by two independent reviewers (C.S. and M.P.). After exclusion of studies deemed irrelevant, the remaining articles were accessed in full and those fulfilling the eligibility criteria were included in the study. Disagreements were resolved by consensus. The reference lists of systematic reviews and retrieved articles were also searched for additional papers, and no language restrictions were applied.

### Study selection and data collection

Two authors (V.K. and C.S.) assessed independently the obtained records for eligibility. Agreement regarding potential relevance was reached by consensus. Full‐text copies of the relevant papers were obtained, and another set of reviewers (D.M.T., V.K., I.B. and M.P.) independently extracted the items of interest using prepiloted forms, agreed upon by all authors. Inconsistencies were discussed by the reviewers until consensus was reached or by discussion with a third author (W.P.M.). If more than one study was published on the same cohort with identical endpoints, the report containing the most comprehensive information on the population was included to avoid overlapping data (Appendix S1). When the information of interest was not provided, the authors of the original studies were contacted via email, using prepiloted forms that included the requested data.

Prespecified items for which data were sought were: name of first author, date of publication, location of study, published protocol (yes/no), funding (yes/no), mean age and body mass index of participants, parity, type of infertility, smoking (pack/years), alcohol consumption (glasses per week), number of participants in each treatment arm, oxygen pressure (low ≤ 6% or atmospheric) in which blastocysts were cultured, number of congenital anomalies in each arm, gestational age at delivery, birth weight, gender, healthy neonate at home and number of perinatal deaths in each arm. Estimates for the contingency table were obtained from the reported risk ratio (RR) using commonly used formulas in the meta‐analysis literature^37^.

### Risk of bias

Two sets of authors (W.P.M., D.M.T. and V.K., I.B.) assessed independently the risk of bias of eligible studies. For RCTs, the risk of bias was assessed using the risk of bias (RoB) 2 tool^38^. RCTs were classified as having low risk of bias, risk of bias of some concerns or high risk of bias. The ROBINS‐I tool was used to assess the risk of bias within each non‐randomized study^39^. The non‐randomized observational studies were classified as having low, moderate, serious or critical risk of bias. Regarding RCTs, blinding was not considered as a likely factor to influence the risk of performance and detection bias for the reproductive outcome^40^. Visualization of the final assessments was performed in line with the common practice of presenting bar charts for the distribution of the judgments in each domain^41^.

### Statistical analysis

#### Summary measures and assessment of heterogeneity

Continuous variables were presented as mean ± SD, median (interquartile range) or median (range). RRs along with their 95% CIs comparing blastocyst‐ *vs* cleavage‐stage embryo transfer were provided for all outcomes. Pairwise meta‐analysis was performed by fitting a random‐effects (DerSimonian–Laird) model, as the true effect size should not be assumed to be the same across studies, and this model incorporated the observed heterogeneity among studies, obtaining more conservative CIs^37^. Reporting was separate for randomized and non‐randomized studies.

Interstudy heterogeneity was assessed by calculating the Cochran's Q statistic and the inconsistency index (*I* ^*2*^), with values > 50% indicating at least substantial heterogeneity^42^.

#### Small‐study effects

As a test for potential small‐study effects, funnel plots for all outcomes are presented^43^. For plots including at least 10 studies, formal testing of asymmetry was also performed (Egger's test; *P* < 0.1 indicating significance)^37^, ^43^. When funnel plot asymmetry was detected, contour‐enhanced funnel plots were created to investigate potential reasons for the observed asymmetry^37^. If publication bias was considered a plausible explanation for the asymmetry and substantial heterogeneity was absent^37^, the non‐parametric trim‐and‐fill method was employed to correct for it^44^.

#### Subgroup analysis

Considering recent data on the potential impact of oxygen concentration during embryo culture on resulting pregnancy outcome^45^, ^46^, a subgroup analysis by oxygen pressure during embryo culture was planned at protocol stage.

A *post‐hoc* subgroup analysis stratified by plurality of pregnancies resulting in live births (i.e. liveborn singletons *vs* multiples) was conducted. *P*‐values < 0.1 indicated significant between‐subgroup differences.

#### Network meta‐analysis

With respect to all outcomes, the following arms were compared within a frequentist network meta‐analysis framework: fresh‐blastocyst, fresh‐cleavage, frozen‐blastocyst and frozen‐cleavage embryo transfers. Network geometry was visualized^47^. Global and local tests for inconsistency were applied to evaluate for transitivity^48^. The global approach for overall inconsistency was computed according to the type of between‐treatment comparison for all cases, and the values were then used to test for global linearity using the Wald test^49^, ^50^. The local test was based on the node‐splitting method by Dias *et al*.^51^, implementing the symmetrical alternative proposed by White^50^. Log‐transformed RRs were used as effect measures and were subsequently back‐transformed to RRs for easier interpretation of results. Pooled RRs in each comparison set and pooled overall RRs were visualized via network forest and interval plots. Cumulative rankings for identifying superiority among the four arms, in terms of all investigated outcomes, were calculated and were then visualized using rankograms^52^. Treatment was considered superior if it had a higher cumulative probability of decreasing the risk of the investigated outcome, except for the healthy neonate outcome. If adequate data to create connected networks were available, separate *post‐hoc* network meta‐analyses of data on liveborn singletons and multiples were performed.

Statistical significance was set at a two‐tailed *P*‐value < 0.05. All analyses were conducted using Stata version 13 (StataCorp. LLP, College Station, TX, USA) using the metan and network packages.

### Certainty of evidence

The certainty of evidence was evaluated using the Grading of Recommendations, Assessment, Development and Evaluations (GRADE) approach, which takes into consideration study limitations, indirectness, imprecision, inconsistency and publication bias^53^, ^54^.

## RESULTS

### Study selection

**Figure 1:**
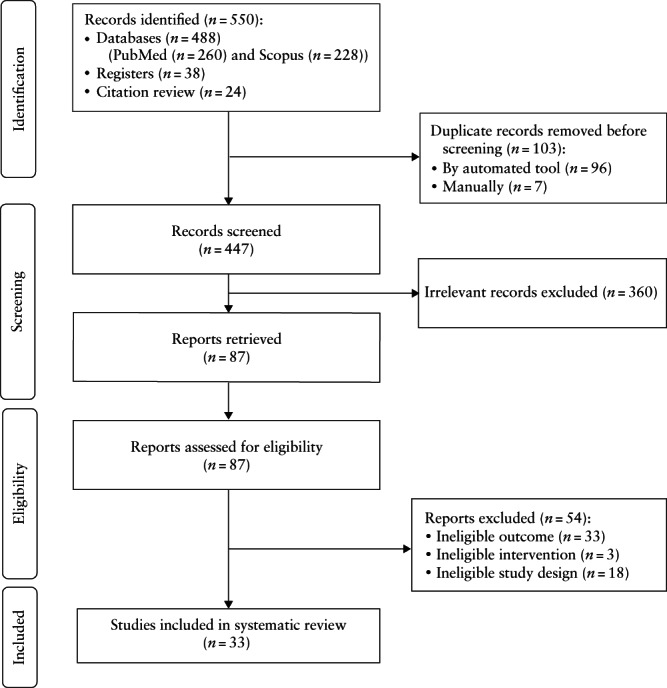
PRISMA flowchart summarizing inclusion in systematic review and meta‐analysis of studies reporting on incidence of any congenital anomaly and pregnancy outcome following cleavage‐ or blastocyst‐stage embryo transfer.

Through the literature search, 550 records were retrieved initially. After screening for relevance, 87 were assessed for eligibility and accessed in full. Following full‐text assessment, 54 studies were excluded (Table S1). The reasons for exclusion were ineligible outcome (33 studies), intervention/comparator (three studies) or study design (11 narrative/systematic reviews and seven retrospective observational studies with < 100 live births in at least one group). The remaining 33 studies (two RCTs and 31 observational studies) were included in this review^10^, ^11^, ^12^, ^14^, ^15^, ^16^, ^17^, ^18^, ^22^, ^23^, ^24^, ^26^, ^27^, ^29^, ^30^, ^55^, ^56^, ^57^, ^58^, ^59^, ^60^, ^61^, ^62^, ^63^, ^64^, ^65^, ^66^, ^67^, ^68^, ^69^, ^70^, ^71^, ^72^. The search is summarized in the PRISMA flowchart (Figure 1).

### Study characteristics and risk of bias

**Figure 2:**
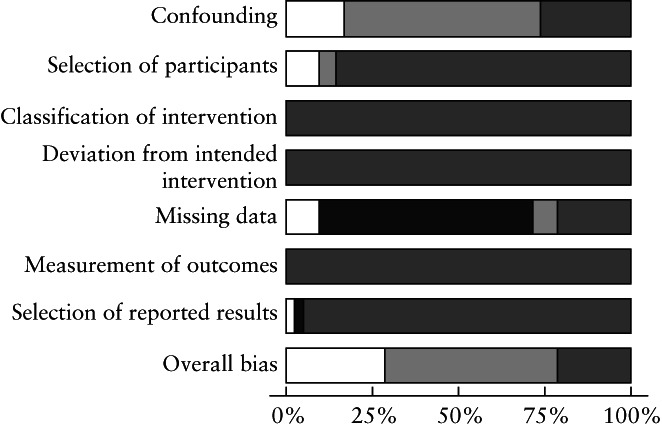
Risk‐of‐bias assessment of observational studies included in systematic review. Risk of bias: 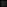, low; 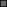, moderate; 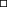, serious; 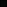, no information.

The main characteristics and assessment for risk of bias of the 33 included studies are presented in Tables 1 and S2, respectively. Of the two RCTs^26^, ^27^, one was deemed to be of low and the other one of high risk of bias. Of the 31 observational studies^10^, ^11^, ^12^, ^14^, ^15^, ^16^, ^17^, ^18^, ^22^, ^23^, ^24^, ^29^, ^30^, ^55^, ^56^, ^57^, ^58^, ^59^, ^60^, ^61^, ^62^, ^63^, ^64^, ^65^, ^66^, ^67^, ^68^, ^69^, ^70^, ^71^, ^72^, six were deemed to be of low, 16 of moderate and nine of serious risk of bias. As shown in Figure 2, issues were found predominantly in the confounding domain. Studies with a potential major overlap of data were not included in the same analysis (Appendix S1).

**Table 1:** Characteristics of 33 studies included in systematic review

			Live delivery						Maternal age (years)	
Study	Country	Study design	Singleton	Multiple	Live infant	Fresh/frozen cycle	Cryopreservation method	Day of blastocyst transfer	Blastocyst	Cleavage
Chambers (2015)^14^	Australia	Cohort	43 952	3418	50 788^h^	Both	Vitrification (67.1% of blastocysts; 6.1% of cleavage‐stage embryos)	5/6	NA	NA
Dar (2013)^55^	Canada	Cohort	12 712^a^	0	12 712^i^	Fresh	NA	5/6	33.8	34.1
De Vos (2015)^56^	Belgium	Cohort	2098	0	2098	Fresh	NA	5	30.7 ± 3.9	33.0 ± 4.2
Fang (2018)^57^	China	Cohort	1092	0	1092	Frozen	Vitrification	5/6	38.8 ± 1.9	38.8 ± 1.6
Fernando (2012)^15^	Australia	Cohort	4202	0	4202	Both	NA	5/6	33.6 ± 4.1	34 ± 4.1
Ginström Ernstad (2016)^10^	Sweden	Cohort	30 566	0	30 566	Both	Vitrification and slow freezing for blastocysts and slow freezing for cleavage‐stage embryos	5/6	NA	NA
Ginström Ernstad (2019)^58^	Sweden, Denmark	Cohort	16 242^b^	0	16 242^b^	Both	Vitrification for blastocysts and slow freezing for cleavage‐stage embryos	NA	Fresh: 34 ± 4.3; Frozen: 34.7 ± 4.2	34.8 ± 4.1
Hatirnaz (2017)^26^	Turkey	RCT	63	5	73	NA	NA	5	30.4 ± 4.3	29.4 ± 4.2
Hattori (2019)^59^	Japan	Cohort	2641	106	2853	Both	NA	NA	35.6 ± 3.9	35.6 ± 4.0
Huang (2020)^22^	China	Cohort	930	0	930	Frozen	Vitrification	5/6/7^q^	32 (29–34)	Day 5: 31 (28–34); Day 6: 32 (29–35); Day 7: 31 (29–34)
Ishihara (2014)^60^	Japan	Cohort	48 158	0	48 158^j^	Both	NA	NA	Fresh: 35.8 ± 4.3; Frozen: 36.0 ± 4.2	Fresh: 37.0 ± 4.6; Frozen: 36.3 ± 4.7
Källén (2010)^11^	Sweden	Cohort	11 590	1136 (1125 twins and 11 triplets)	13 873	NA	NA	NA	NA	NA
Kalra (2012)^12^	USA	Cohort	47 133^c^	10 209^f^	67 551^k^	Fresh	NA	5/6	33.5 ± 4.0	34.5 ± 4.1
Kato (2012)^61^	Japan	Cohort	6623	0	6623	Both	Vitrification	5/6/7	Fresh SET 37.9 (4.7); Frozen SET 38.1 (4.2)	
Kausche (2001)^62^	Australia	Cohort	1740	428 (390 twins and 38 triplets)	2634^l^	Both	NA	5/6/7	NA	NA
Levi‐Setti (2018)^27^	Italy	RCT	77	41	159	Fresh	NA	5/6	33.5 ± 2.9	33.4 ± 2.9
Li (2017)^63^	China	Cohort	652	303	1258	Fresh	NA	5	29.3 ± 4.0	29.7 ± 4.1
Litzky (2018)^30^	USA	Cohort	124 154	0	124 154	Fresh	NA	5/6	33.72 (4.18)	
Long (2020)^23^	China	Cohort	5345	1023	7391^m^	Frozen	NA	NA	NA	NA
Marconi (2019)^16^	UK	Cohort	67 147	0	67 147	Fresh	NA	NA	NA	NA
Martin (2012)^64^	France	Cohort	1547^d^	0	1547^d^	Fresh	NA	5/6	32.3 ± 3.9	32.6 ± 3.8
Maxwell (2015)^65^	USA	Cohort	2392^e^	0	2392^e^	Fresh	NA	5/6	35.5 ± 4.2	37.9 ± 3.9
Milki (2003)^66^	USA	Cohort	279	90	459	NA	NA	5	NA	NA
Oron (2015)^17^	Canada	Matched case–control	318	0	318^n^	Fresh	NA	5	33.3 ± 3.6	33.4 ± 4.0
Sazonova (2011)^67^	Sweden	Cohort	8941	0	8941	Fresh	NA	5	NA	NA
Schwärzler (2004)^68^	Austria	Cohort	274	111 (107 twins and four triplets)	500	Fresh	NA	5	31 ± 4.2	34 ± 4.5
Shi (2019)^18^	China	Cohort	8995	3577 (3576 twins and one triplets)	16 150^o^	Both	Vitrification	5/6	NA	NA
Sotiroska (2015)^69^	North Macedonia	Cohort	381	236 (217 twins and 19 triplets)	872	Fresh	NA	5	< 36 years: 29.4 ± 3.1; > 36 years: 38.5 ± 2.1	< 36 years: 30.0 ± 3.3; > 36 years: 38.9 ± 2.6
Spangmose (2020)^24^	Denmark, Norway, Sweden	Cohort	56 557	0	56 557	Fresh	NA	NA	34 ± 4.3	34 ± 4.3
Wang (2010)^70^	Australia	Cohort	NA	NA	29 533^p^	Both	NA	NA	NA	NA
Wikland (2010)^71^	Sweden	Cohort	496	0	496	Both	Vitrification for blastocysts and slow freezing for cleavage‐stage embryos	5/6	Fresh: 34.7 (22.0–44.0); Frozen: 35.4 (26.3–45.3)^r^	Frozen: 35.6 (24.9–43.5)^r^
Zhou (2018)^72^	China	Cohort	5107	1992	9091	Frozen	Vitrification of cleavage‐stage embryos	5	NA	NA
Zhu (2018)^29^	China	Cohort	12 625	2193^g^	17 015^g^	Both	Vitrification	5/6	NA	NA

Data are given as *n*, mean ± SD or median (interquartile range).

Live delivery was defined as single birth event of one or more live neonates.

Multiple delivery refers to delivery of twins, unless stated otherwise.

Only first author is given for each study.

^a^ Data on preterm delivery available for 12 636 deliveries.

^b^ In order to avoid potential population overlap between Ginström Ernstad (2019)^58^ and Spangmose (2020)^24^ studies, the 4469 deliveries/infants from the fresh‐blastocyst‐transfer group of the Ginström Ernstad (2019)^58^ study were excluded from pairwise meta‐analysis; hence 11 773 deliveries/infants were included.

^c^ Data on preterm delivery available for 47 094 deliveries.

^d^ All data available for 1183 deliveries/liveborn infants.

^e^ All data available for 1861 deliveries/liveborn infants.

^f^ Data on preterm delivery available for 10 192 deliveries.

^g^ Data on plurality and congenital anomaly stratified by plurality available for four infants.

^h^ All data available for 49 780 liveborn infants.

^i^ Data on low birth weight available for 12 094 liveborn infants.

^j^ Data on gender available for 47 900 liveborn infants.

^k^ Data on low birth weight available for 66 807 liveborn infants.

^l^ Data on gender available for 2631 liveborn infants.

^m^ Data on low birth weight available for 6368 liveborn infants.

^n^ Data on gender available for 231 liveborn infants.

^o^ Data on low birth weight available for 16 020 liveborn infants.

^p^ Calculated based on provided live‐birth‐rate and transfer‐cycle data.

^q^ Separate data for days 5, 6 and 7.

^r^ Age is reported as median (range).

NA, not available/not applicable; RCT, randomized controlled trial; SET, single embryo transfer.

### Synthesis of results

#### Pairwise meta‐analysis

**Figure 3:**
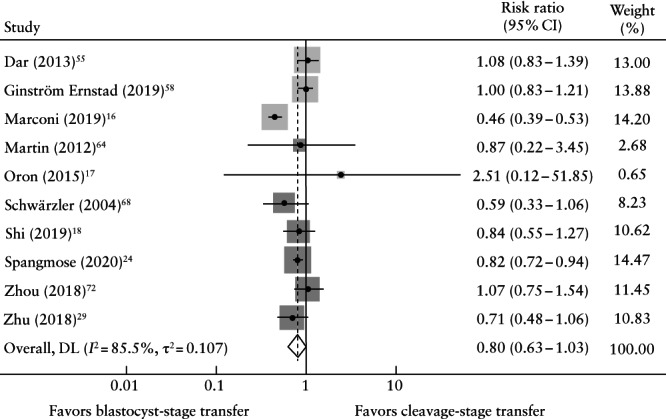
Pairwise meta‐analysis forest plot showing risk ratio with 95% CI for any congenital anomaly, according to whether pregnancy was conceived following cleavage‐ or blastocyst‐stage transfer. Weights are from random‐effects model; continuity correction was applied to studies with zero cells. Only first author's name is given for each study. DL, DerSimonian–Laird random‐effects meta‐analysis model.

There was no significant difference in the risk of any congenital anomaly following cleavage‐ *vs* blastocyst‐transfer (RR, 0.803 (95% CI, 0.626–1.030), 10 studies^16^, ^17^, ^18^, ^24^, ^29^, ^55^, ^58^, ^64^, ^68^, ^72^, *n* = 192 442 liveborn neonates, *I* ^2^ = 85.5%; Figure 3). Only one RCT reporting on the rate of congenital anomaly was identified (blastocyst (*n* = 2 (2.6%)) *vs* cleavage (*n* = 0 (0%)) liveborn neonates; *P* = 0.233)^27^. All studies provided data on liveborn neonates, whereas only one study reported on terminated cases, finding no significant difference in the rate of congenital anomaly (blastocyst (*n* = 21 (0.4% of clinical pregnancies)) *vs* cleavage (*n* = 51 (0.5% of clinical pregnancies)))^18^.

There was no significant difference in the rates of PTD (1.024 (95% CI, 0.920–1.139), 18 studies^12^, ^14^, ^15^, ^16^, ^17^, ^18^, ^23^, ^24^, ^55^, ^56^, ^57^, ^58^, ^60^, ^64^, ^65^, ^68^, ^69^, ^72^, *n* = 338 722 live deliveries, *I* ^2^ = 95.0%), LBW (0.953 (95% CI, 0.867–1.048), 17 studies^12^, ^14^, ^15^, ^16^, ^17^, ^18^, ^22^, ^23^, ^24^, ^30^, ^55^, ^56^, ^57^, ^58^, ^60^, ^68^, ^72^, *n* = 477 089 liveborn neonates, *I* ^2^ = 95.2%), perinatal death (0.974 (95% CI, 0.775–1.224), six studies^24^, ^55^, ^58^, ^64^, ^69^, ^72^, *n* = 92 188, *I* ^2^ = 0%) or healthy neonate (1.049 (95% CI, 0.999–1.101), two studies^16^, ^70^, *n* = 96 680 neonates, *I* ^2^ = 96.2%) following blastocyst‐ *vs* cleavage‐stage transfer (Figure S1a–c,e). Only one RCT^26^ reported on perinatal mortality, indicating that there was no significant difference between the interventions (cleavage (*n* = 2 (2.1%)) *vs* blastocyst (*n* = 4 (4.2%)); *P* = 0.407). The calculation of these rates was based on the number of embryo transfers in the original study. No significant difference was found when using the number of clinical pregnancies as the denominator (cleavage (*n* = 2 (4.5%)) *vs* blastocyst (*n* = 4 (9.5%)); *P* = 0.428 (Fisher's exact test)).

The probability of a male neonate was slightly but statistically significantly higher following blastocyst transfer (1.072 (95% CI, 1.055–1.088), 18 studies^15^, ^16^, ^17^, ^18^, ^22^, ^24^, ^56^, ^57^, ^58^, ^59^, ^60^, ^62^, ^64^, ^65^, ^66^, ^68^, ^69^, ^72^, *n* = 227 530 neonates, *I* ^2^ = 32.7%; Figure S1d). One RCT^27^ reported on gender of neonates, demonstrating no significant difference in male‐to‐female ratios between the two modalities.

#### Subgroup pairwise meta‐analysis

Two studies^71^, ^72^ reported on the oxygen conditions during culture, and in both studies, it was set at 5%. Due to the lack of variability in oxygen conditions, this perprotocol subgroup analysis was not performed.

No significant between‐subgroup differences (singleton *vs* multiple pregnancy) were observed with respect to congenital anomaly, PTD, LBW and gender of the neonate (Figure S2a–c,e). One RCT^27^ reporting on gender of the neonate demonstrated no significant difference in male/female ratios between the two modalities in both singletons (*P* = 0.821) and twins (*P* = 0.826). When considering perinatal mortality, only the subpopulation of singletons could be analyzed (Figure S2d), while for the healthy neonate outcome, no such subgroup analysis could be conducted. The results of the analysis focusing on singletons only were similar to those of the analysis of the overall population including both singleton and multiple pregnancy.

#### Network meta‐analysis

Five studies^10^, ^18^, ^29^, ^60^, ^70^ were stratified by both the use of cryopreservation (i.e. fresh *vs* frozen cycle) and embryo developmental stage at transfer (i.e. blastocyst‐ *vs* cleavage‐stage transfer) and led to connected networks (Figure S3 **)**. Node sizes indicated fewer studies reporting on frozen cycles. The body of evidence was consistent in all network meta‐analyses, except for the meta‐analysis on any congenital anomaly (Figures S4–e and S5a–e).

**Figure 4:**
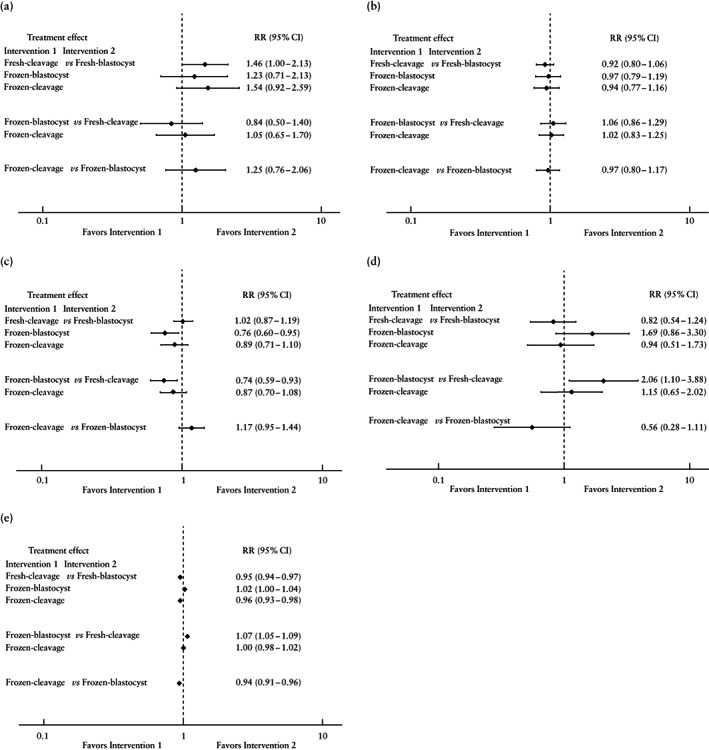
Network meta‐analysis interval plots showing risk ratio (RR) of any congenital anomaly (a), preterm delivery (< 37 weeks) (b), low birth weight (< 2500 g) (c), perinatal death (d) and male neonate (e) following fresh‐cleavage, fresh‐blastocyst, frozen‐cleavage or frozen‐blastocyst transfer.

Between‐arm comparisons indicated no significant difference in the risk of any congenital anomaly (*n* = 116 725 liveborn infants, six studies^10^, ^16^, ^17^, ^29^, ^64^, ^68^; Figures 4a, S5a). The network meta‐analysis of singletons only demonstrated similar results, and the body of evidence for this specific population was consistent (*n* = 99 214 liveborn infants, four studies^10^, ^16^, ^17^, ^64^) (Figure S6a). Data on liveborn infants from multiple pregnancy were inadequate to create a connected network.

Between‐group comparisons suggested no significant difference in the risk of PTD (*n* = 227 553 live deliveries, 12 studies^10^, ^12^, ^16^, ^17^, ^18^, ^23^, ^57^, ^60^, ^64^, ^65^, ^68^, ^69^; Figures 4b, S5b). The same applied when evaluating singletons only (*n* = 207 069 deliveries, 11 studies^10^, ^12^, ^16^, ^17^, ^18^, ^57^, ^60^, ^64^, ^65^, ^68^, ^69^; Figure S6b). When considering only multiple pregnancy, fresh‐cleavage transfer reduced significantly the risk of PTD compared with frozen‐cleavage, fresh‐ and frozen‐blastocyst transfers (*n* = 14 116 deliveries, four studies^12^, ^18^, ^68^, ^69^; Figure S7a). Additionally, frozen‐blastocyst transfer increased significantly the risk for PTD compared with fresh‐blastocyst transfer in multiple pregnancy.

The risk of LBW appeared to be significantly lower following frozen‐blastocyst transfer compared with both fresh‐blastocyst (RR, 0.76 (95% CI, 0.60–0.95)) and fresh‐cleavage (RR, 0.74 (95% CI, 0.59–0.93)) transfers. The other comparisons did not indicate significant differences among interventions (*n* = 252 098 liveborn infants, 12 studies^10^, ^12^, ^16^, ^17^, ^18^, ^22^, ^23^, ^55^, ^56^, ^57^, ^60^, ^68^; Figures 4c, S5c). The results did not change substantially when only singletons were analyzed (*n* = 218 313 liveborn infants, 11 studies^10^, ^12^, ^16^, ^17^, ^18^, ^22^, ^55^, ^56^, ^57^, ^60^, ^68^; Figure S6c). Both frozen‐blastocyst and fresh‐cleavage transfer methods slightly but significantly decreased the risk of LBW in multiple pregnancy when compared with fresh‐blastocyst transfer (*n* = 27 417 liveborn infants, three studies^12^, ^18^, ^68^; Figure S7b).

Frozen‐blastocyst transfer increased significantly the risk of perinatal death when compared with fresh‐cleavage transfer (RR, 2.06 (95% CI, 1.10–3.88)). All other comparisons showed no significant differences among the interventions (*n* = 45 333 infants/fetuses, four studies^10^, ^55^, ^64^, ^69^; Figures 4d, S5d). The results did not change when only singletons were analyzed (*n* = 44 461 infants/fetuses, three studies^10^, ^55^, ^64^; Figure S6d), whereas data on multiple pregnancy were inadequate to create a connected network.

Both fresh‐cleavage (RR, 0.95 (95% CI, 0.94–0.97)) and frozen‐cleavage (RR, 0.96 (95% CI, 0.93–0.98)) embryo transfer methods slightly but significantly reduced the probability of a male neonate compared with fresh‐blastocyst transfer (*n* = 154 546 liveborn infants, 11 studies^10^, ^16^, ^17^, ^22^, ^56^, ^60^, ^63^, ^64^, ^65^, ^68^, ^69^; Figures 4e, S5e). The frozen‐cleavage modality led to a significantly decreased probability of a male neonate compared with frozen‐blastocyst transfer (RR, 0.94 (95% CI, 0.91–0.96)), while the latter increased this probability when compared with fresh‐cleavage transfer (RR, 1.07 (95% CI, 1.05–1.09)). The analysis on singletons only provided similar results (*n* = 152 568, nine studies^10^, ^16^, ^17^, ^22^, ^56^, ^60^, ^63^, ^64^, ^65^; Figure S6e). Data on multiple pregnancy were inadequate to create a connected network. Due to the very small number of studies reporting on the healthy neonate outcome (*n* = 2)^16^, ^70^, a network meta‐analysis was not conducted.

#### Ranking of treatments

Relative ranking of treatments with respect to all outcomes (considering both singleton and multiple pregnancy) via calculation of cumulative ranking probabilities and visualization through rankograms is available in Figures S8a–e. Potential superiority was demonstrated by the fresh‐blastocyst arm (followed by the frozen‐blastocyst arm) in reducing the risk of any congenital anomaly in liveborn neonates, fresh‐ and frozen‐cleavage arms in reducing the risk of PTD, frozen‐blastocyst arm (followed by frozen‐cleavage) in decreasing the risk of LBW, fresh‐cleavage arm (followed by frozen‐cleavage) in reducing the risk of perinatal death, and fresh and frozen‐cleavage arms in reducing the probability of higher male births. These findings should be interpreted in combination with the effect estimates reported above.

### Small‐study effects

The funnel plots for all outcomes are presented in Figures S9a–e. The analysis on the healthy neonate outcome included only two studies; therefore, a funnel plot was not created. Visual inspection and the formal test (Egger's regression, when performed) demonstrated no major funnel plot asymmetry with respect to the rest of the investigated outcomes.

### Certainty of evidence

All outcomes were classified as having very‐low certainty of evidence due to limitations of the included studies. Certainty of evidence on the congenital anomaly (95% CI, 0.63–1.03; *I* ^2^ = 85.5%), PTD (95% CI, 0.92–1.14; *I* ^2^ = 95.0%) and LBW (95% CI, 0.87–1.05; *I* ^2^ = 95.2%) outcomes was downgraded further due to both imprecision and inconsistency. Certainty of evidence on the healthy neonate (*I* ^2^ = 96.2%) outcome was downgraded further due to inconsistency, while certainty of evidence on the perinatal death outcome was downgraded further due to imprecision only (95% CI, 0.78–1.22).

## DISCUSSION

### Summary of evidence

The present systematic review demonstrates that there may be little‐to‐no difference in the risk of any congenital anomaly or adverse perinatal outcome in the newborns following blastocyst‐ *vs* cleavage‐stage embryo transfer when the effect of cryopreservation is not taken into account. The network meta‐analysis supported the findings of the pairwise meta‐analysis concerning the primary outcome. When considering the overall included population and singletons only, it further showed a lower risk of LBW in the frozen‐blastocyst compared with the fresh modalities; in multiple pregnancy, the risk was reduced following both frozen‐blastocyst and fresh‐cleavage transfers when compared with fresh‐blastocyst transfer. The fresh‐cleavage modality demonstrated potential superiority in reducing the risk for perinatal death when compared with frozen‐blastocyst transfer. The higher probability of a male neonate following blastocyst transfer was demonstrated by the pairwise and network meta‐analyses. In the multiple‐pregnancy group, fresh‐cleavage transfer reduced significantly the risk of PTD compared with all other transfers, and frozen‐blastocyst transfer increased significantly the risk compared with the fresh‐blastocyst one. All other comparisons revealed non‐significant differences between the interventions.

### Clinical significance of this study with respect to that of previous studies

A considerable number of original studies on the topic^16^, ^18^, ^22^, ^23^, ^24^, ^28^, ^59^ have emerged since the publication of the latest relevant systematic reviews^19^, ^20^, ^21^. A key challenge encountered by former studies was the inability to explore concurrently the effect of developmental stage at the time of transfer and the impact of cryopreservation on the outcome of interest. Two former systematic reviews^19^, ^21^ attempted to address this issue. A recent, more up‐to‐date systematic review has adopted a similar approach with more available data^31^. The present work aimed to evaluate studies that reported on outcomes of interest in a stratified fashion to create connected networks of four interventions and synthesize separately data on multiple pregnancy.

The concurrent consideration of the effect of cryopreservation and the stage of embryo transfer when conducting comparisons is of high clinical value. The freeze‐all strategy potentially results in lower odds of development of ovarian hyperstimulation syndrome and comparable cumulative pregnancy, perinatal and neonatal outcomes when compared with conventional strategies^73^, ^74^, ^75^. Extending *in‐vitro* culture to the blastocyst stage is based primarily on previously reported higher live‐birth rates, especially in good‐prognosis patients^2^, ^6^, but such an effect has been recently questioned^20^. Pregnancy outcomes may be altered when combining certain freezing protocols with specific developmental stages^2^, ^76^; this observation reinforced the need for the network comparisons of the present meta‐analysis.

### Interpretation and comparison with other systematic reviews

In accordance with most former systematic reviews^6^, ^20^, ^31^, we found no significant difference in the risk of any congenital anomaly in newborns conceived via blastocyst‐ *vs* cleavage‐stage transfer.

With respect to PTD, previous meta‐analyses have indicated a slightly higher risk in the blastocyst‐transfer group^6^, ^9^, ^19^, ^20^, ^21^. Former studies have attributed this association to potential genetic or epigenetic changes in trophodermal cells during the extended culture^13^, ^76^, ^77^, defective implantation^6^ or the association of male gender with preterm birth^9^, ^78^. The most up‐to‐date work^31^ demonstrated a higher risk of VPTD following fresh‐blastocyst compared with fresh‐cleavage transfer^21^, ^31^. Similar findings on PTD were reported after synthesis of adjusted data^31^; this evidence was derived from subgroup analyses (frozen‐blastocyst *vs* frozen‐cleavage, and fresh‐blastocyst *vs* fresh‐cleavage), yet the overall comparison indicated no significant difference, similar to this study^31^. Notably, the data of most reviews applied to singleton pregnancy, while the present work considers both singleton and multiple pregnancy. However, the subgroup analyses by plurality of pregnancy indicated no significant between‐subgroup differences. When considering singletons only, the results were in accordance with those of the overall mixed population.

Although the pairwise analysis demonstrated no significant difference in the risk of LBW, in agreement with the findings of the majority of previous papers^9^, ^20^, ^21^, ^31^, a network comparison demonstrated that the risk was significantly lower following frozen‐blastocyst *vs* both fresh‐transfer modalities. In contrast, a slightly higher risk associated with the blastocyst stage was observed by one systematic review, which lacked data on frozen cycles^6^, while in another, no differences were noted between the subsets^23^. This may be related to certain reported advantages of frozen‐ over fresh‐embryo transfer^2^, ^20^, ^79^, ^80^, although current evidence suggests that the potential effect of frozen transfer on the long‐term health of newborns needs further investigation^81^, ^82^.

We also noted that fresh‐cleavage transfer was associated with a lower risk of perinatal death compared with the frozen‐blastocyst modality. A higher risk for perinatal mortality in the blastocyst group was indicated by one systematic review^20^, supporting the network findings of this study. When considering the comparison between any blastocyst and any cleavage technique, however, no significant differences have been observed between groups^31^.

A previous systematic review focusing on fresh‐embryo transfer only reported a potential association between blastocyst transfer and offspring sex ratio skewed in favor of males^83^. This was supported by the findings of our work. Male embryos have been reported to develop more rapidly in animal species, increasing their probability of being selected for transfer at the blastocyst stage^83^.

Finally, the performed relative ranking of treatments with respect to all outcomes revealed the potential superiority of blastocyst transfer in reducing the risk of any congenital anomaly, cleavage transfer in reducing the risk for PTD, perinatal death and higher incidence of live male births, and frozen modalities in decreasing the risk of LBW. Nevertheless, interpretations of findings may not always be feasible based on current available evidence and should be interpreted in combination with the reported effect estimates.

### Strengths and limitations

This is the first systematic review to investigate the effect of both the stage of embryo transfer and cryopreservation on the rate of any congenital anomaly and perinatal outcome by directly and indirectly comparing outcomes of all possible treatment combinations within a frequentist network meta‐analysis. Although a meta‐analysis of RCTs would be preferred, it could not be performed given the limited availability of such studies; hence, the meta‐analysis included observational studies with a sample of at least 100 live births per group. Few of the 33 included studies were judged to have a low overall risk of bias, while all outcomes were assessed as having very‐low certainty of evidence and were downgraded by the limitations of the included studies, inconsistency and/or imprecision. Inconsistency was noted in the congenital anomaly network; therefore, findings should be interpreted with caution. Due to the lack of individual patient data or inconsistent aggregate data reporting, we were unable to account for potential confounders like freezing methods, culture conditions or day of blastocyst transfer.

### Conclusion

Current very‐low certainty of evidence shows that there may be little‐to‐no difference in the risk of any congenital anomaly and adverse perinatal outcome following blastocyst‐ *vs* cleavage‐stage embryo transfer, though there might be a slightly increased probability of a male newborn following blastocyst transfer. Network meta‐analysis demonstrated a significantly lower risk of LBW following frozen‐blastocyst transfer *vs* both fresh modalities, a significantly lower risk of perinatal death following fresh‐cleavage *vs* frozen‐blastocyst transfer and a significantly lower risk for PTD of multiple pregnancies following fresh‐cleavage transfer compared with all other modalities. High‐quality RCTs with separate data on fresh and frozen cycles, and explicit, consistent reporting of culture conditions and freezing methods are mandatory in future research. Individual participant data meta‐analyses may account for potential confounders and resolve the substantial inconsistency associated with current aggregate data approaches.

## Supporting information


**Table S1** Excluded studies with reasons
**Table S2** Risk of bias summary: authors' judgements about each risk‐of‐bias item of the 33 included studies
**Appendix S1** Studies with potentially duplicate populations after comparisons of study centers and study periods
**Figure S1** Pairwise meta‐analysis forest plots showing risk ratios for preterm delivery (< 37 weeks) (a), low birth weight (< 2500 g) (b), perinatal death (c), male neonate (d) and healthy neonate (e). DL, DerSimonian–Laird random‐effects meta‐analysis model.
**Figure S2** Pairwise meta‐analysis forest plots showing risk ratios in liveborn singleton and multiple pregnancies for any congenital anomaly (a), preterm delivery (< 37 weeks) (b), low birth weight (< 2500 g) (c), perinatal death (d) and male neonate (e) following blastocyst *vs* cleavage transfer. DL, DerSimonian–Laird random‐effects meta‐analysis model.
**Figure S3** Network geometry. Size of nodes representing four embryo transfer arms is indicative of the number of included studies per arm, while thickness of lines is indicative of the amount of data. Nodes representing studies on frozen cycles are smaller.
**Figure S4** Network sidesplitting of nodes (local test on inconsistency), examining differences between direct and indirect evidence (measure of effect: risk ratio, log scale) for any congenital anomaly (a), preterm delivery (< 37 weeks) (b), low birth weight (< 2500 g) (c), perinatal death (d) and male neonate (e). A symmetrical alternative of the Dias *et al*.^51^ method was employed.
**Figure S5** Network meta‐analysis forest plots for all pregnancies showing risk ratio for any congenital anomaly (a), preterm delivery (< 37 weeks) (b), low birth weight (< 2500 g) (c), perinatal death (d) and male neonate (e).
**Figure S6** Network meta‐analyses forest and interval plots for singleton pregnancies showing risk ratio for any congenital anomaly (a), preterm delivery (< 37 weeks), low birth weight (< 2500 g) (c), perinatal death (d) and male neonate (e).
**Figure S7** Network meta‐analyses forest and interval plots for multiple pregnancies showing risk ratio for preterm delivery (< 37 weeks) (a) and low birth weight (< 2500 g) (b). Only data on preterm delivery and low birth weight were sufficient to create connected networks.
**Figure S8** Network rankograms and cumulative ranking probabilities for identifying superiority of treatments in reducing the risk for any congenital anomaly (a), preterm delivery (< 37 weeks) (b), low birth weight (< 2500 g) (c), perinatal death (d) and male neonate (e).
**Figure S9** Funnel plots of meta‐analyses on any congenital anomaly (a), preterm delivery (< 37 weeks) (b), low birth weight (< 2500 g) (c), perinatal death (d) and male neonate (e). Effect size (log risk‐ratio scale) is plotted against the corresponding standard error. Results of Egger's regression test (*P*‐value) for funnel plot asymmetry are presented at the bottom left part of each figure.

## Data Availability

The data that support the findings of this study are available from the corresponding author upon reasonable request.
