# Supplementary material for: Congenital anomaly and perinatal outcome following blastocyst‐ vs cleavage‐stage embryo transfer: systematic review and network meta‐analysis
Source: Ultrasound Obstet Gynecol. 2023 Jan 3;61(1):12–25. doi: 10.1002/uog.26019 (PMC10107888; doi:10.1002/uog.26019)
Supplement: Supplementary file 1 — Table S1 Excluded studies with reasons Table S2 Risk of bias summary: authors' judgements about each risk‐of‐bias item of the 33 included studies Appendix S1 Studies with potentially duplicate populations after comparisons of study centers and study periods Figure S1 Pairwise meta‐analysis forest plots showing risk ratios for preterm delivery (< 37 weeks) (a), low birth weight (< 2500 g) (b), perinatal death (c), male neonate (d) and healthy neonate (e). DL, DerSimonian–Laird random‐effects meta‐analysis model. Figure S2 Pairwise meta‐analysis forest plots showing risk ratios in liveborn singleton and multiple pregnancies for any congenital anomaly (a), preterm delivery (< 37 weeks) (b), low birth weight (< 2500 g) (c), perinatal death (d) and male neonate (e) following blastocyst vs cleavage transfer. DL, DerSimonian–Laird random‐effects meta‐analysis model. Figure S3 Network geometry. Size of nodes representing four embryo transfer arms is indicative of the number of included studies per arm, while thickness of lines is indicative of the amount of data. Nodes representing studies on frozen cycles are smaller. Figure S4 Network sidesplitting of nodes (local test on inconsistency), examining differences between direct and indirect evidence (measure of effect: risk ratio, log scale) for any congenital anomaly (a), preterm delivery (< 37 weeks) (b), low birth weight (< 2500 g) (c), perinatal death (d) and male neonate (e). A symmetrical alternative of the Dias et al. 51 method was employed. Figure S5 Network meta‐analysis forest plots for all pregnancies showing risk ratio for any congenital anomaly (a), preterm delivery (< 37 weeks) (b), low birth weight (< 2500 g) (c), perinatal death (d) and male neonate (e). Figure S6 Network meta‐analyses forest and interval plots for singleton pregnancies showing risk ratio for any congenital anomaly (a), preterm delivery (< 37 weeks), low birth weight (< 2500 g) (c), perinatal death (d) and male neonate (e). Figure S7 Network m [file UOG-61-12-s001.pdf]

Supplementary Table 1 (Table S1). Excluded studies with reasons (these studies are not cited in the main text).

Study	Journal	Reason for exclusion
1. Aziminekoo 2015 https://doi.org/10.3109/09513590.2015.1056141	Gynecol Endocrinol	Not assessing outcomes of interest
2. Bouillon 2017 https://doi.org/10.1016/j.rbmo.2017.04.009	Reprod Biomed Online	Not assessing outcomes of interest
3. Bungun 2003 https://doi.org/10.1016/s1472-6483(10)61736-1	Reprod Biomed Online	Not assessing outcomes of interest
4. Chang 2009 https://doi.org/10.1016/j.fertnstert.2008.03.066	Fertil Steril	Review
5. Chen 2015 https://doi.org/10.3109/09513590.2015.1062858	Gynecol Endocrinol	Not assessing outcomes of interest
6. Coscun 2000 https://doi.org/10.1093/humrep/15.9.1947	Hum Reprod	Not assessing outcomes of interest
7. Dar 2014 doi: 10.1093/humupd/dmu001	Hum Reprod Update.	Review
8. De Vos 2016 doi: 10.1093/humrep/dew219	Hum Reprod	Ineligible outcome
9. De Vos 2020 doi: 10.1007/s10815-019-01641-4	J Assist Reprod Genet	<100 births in at least one cohort
10. Du 2018 10.1093/humrep/dey092	Hum Reprod	Not assessing outcomes of interest
11. Eftekhar 2020 doi: 10.1017/S0967199420000428	Zygote	Not assessing outcomes of interest
12. Emiliani 2003 https://doi.org/10.1093/humrep/deg394	Hum Reprod	Not assessing outcomes of interest
13. Fernández-Shaw 2015 https://doi.org/10.1007/s10815-014-0387-9	J Assist Reprod Genet.	Not assessing outcomes of interest
14. Glujovsky D 2016 10.1002/14651858.CD002118.pub5	Cochrane Database of Systematic Reviews	Review

15. Ho 2018 10.1007/s10815-018-1113-9	J Assist Reprod Genet	Not assessing outcomes of interest
16. Hreinsson 2004 https://doi.org/10.1016/j.ejogrb.2004.06.011	Eur J Obstet Gynecol Reprod Biol	Not assessing outcomes of interest
17. Jwa 2015 Doi: 10.1016/j.fertnstert.2015.03.029	Fertil Steril	Ineligible intervention/ comparator (assisted hatching vs non-assisted hatching)
18. İnal 2021 doi: 10.4274/jtgga.galenos.2021.2020.0171	J Turk Ger Gynecol Assoc	Not assessing outcomes of interest
19. Kaartinen 2015 10.1016/j.rbmo.2014.11.016	Reprod Biomed Online	Not assessing outcomes of interest
20. Kaser 2017 doi.org/10.1093/humrep/dex231	Hum Reprod	Not assessing outcomes of interest
21. Kaur 2014 https://doi.org/10.4103/0974-1208.142481	J Hum Reprod Sci	Not assessing outcomes of interest
22. Kolibianakis 2004 https://doi.org/10.1093/humrep/deh447	Hum Reprod	Not assessing outcomes of interest
23. Korosec 2016 10.1007/s10815-015-0601-4	J Assist Reprod Genet	Not assessing outcomes of interest
24. Le 2021 doi: 10.1016/j.cryobiol.2021.04.003	Cryobiology	Not assessing outcomes of interest
25. Li 2021 doi: 10.1038/s41598-021-87693-y	Sci Rep	Not assessing outcomes of interest
26. Maheshwari 2016 doi.org/10.1016/j.rbmo.2015.09.016	Reprod Biomed Online	Review
27. Maheswari 2013 doi.org/10.1016/j.fertnstert.2013.08.044	Fertil Steril	Review
28. Makinen 2013 doi: 10.1093/humrep/des410	Hum Reprod	<100 births in at least one cohort
29. Marconi 2021 doi:10.1093/humupd/dmab042	Hum Reprod Update	Review
30. Martins 2016 doi.org/10.1093/humrep/dew244	Hum Reprod	Review
31. Martins 2017 doi:10.1002/uog.17327	Ultrasound Obstet Gynecol	Review

32. Montag 2006 doi.org/10.1016/j.ejogrb.2005.08.022	Eur J Obstet Gynecol Reprod Biol	Not assessing outcomes of interest
33. Nakshima 2013 doi:10.1016/j.fertnstert.2012.09.027.	Fertil Steril	Not assessing outcomes of interest
34. Oron 2014 doi: 10.1016/j.ajog.2014.03.018	Am J Obstet Gynecol	<100 births in at least one cohort
35. Pantos 2004 PMID: 14998186	Clin Exp Obstet Gynecol	Not assessing outcomes of interest
36. Papanikolaou 2005 doi.org/10.1093/humrep/dei217	Hum Reprod	Not assessing outcomes of interest
37. Papanikolaou 2006 doi.org/10.1056/NEJMoa053524	N Engl J Med	Not assessing outcomes of interest
38. Pereira 2016 doi: 10.1155/2016/1245210	J Pregn	<100 births in at least one cohort
39. Pinborg 2013 doi.org/10.1016/j.fertnstert.2012.12.001	Fertil Steril	Review
40. Rienzi 2002 doi.org/10.1093/humrep/17.7.1852	Hum Reprod	Not assessing outcomes of interest
41. Rodriguez 2001 doi: 10.1016/S0015-0282(01)02502-X	Fertil Steril	<100 births in at least one cohort
42. SART doi.org/10.1016/j.fertnstert.2018.09.011	Fertil Steril	Review
43. Tatsumi 2017 doi: 10.1093/humrep/dew280	Hum Reprod	Ineligible intervention/ comparator (natural cycle vs letrozole)
44. Torky 2021 doi: 10.5935/1518-0557.20200083	JBRA Assist Reprod	Not assessing outcomes of interest
45. Vajta 2020 doi: 10.1093/humrep/deaa202	Hum Reprod	Not assessing outcomes of interest
46. Van Der Auwera 2002 doi.org/10.1093/humrep/17.6.1507	Hum Reprod	Not assessing outcomes of interest
47. Wang 2017 doi: 10.1186/s12958-017-0255-4	Reprod Biol Endocrinol	Review
48. Wang 2019 doi: 10.1080/09513590.2019.1594762	Gynecol Endocrinol	<100 births in at least one cohort
49. Wei 2017 https://doi.org/10.1186/s13063-017-1993-5	Trials	Not assessing outcomes of interest

50. Yang 2018<br>doi: 10.1093/humrep/dey047	Hum Reprod	Ineligible outcome
51. Zhang HN<br>doi: 10.12659/MSM.928737	Med Sci Monit	Single-Morula Embryo Transfer and Single-Blastocyst Transfer
52. Zhu 2014<br>doi: 10.1093/humrep/det460	Hum Reprod	<100 births in at least one cohort
53. Zhu 2020<br>doi: 10.3389/fphys.2020.00930. eCollection 2020	Front Physiol	Not assessing outcomes of interest
54. Zilberberg E<br>doi: 10.1007/s00404-021-06003-z	Arch Gynecol Obstet	Not assessing outcomes of interest

Supplementary Table 2 (Table S2). Risk of bias summary: authors' judgements about each risk of bias item of the 33 included studies.

Study	Confounding	Selection of participants	Classif. Interv.	Dev. from intended interv.	Missing data	Meas. outcomes	Sel. reported result	Overall bias
1. Chambers 2015	Moderate	Low	Low	Low	Moderate	Low	Low	Moderate
2. Dar 2013	Moderate	Low	Low	Low	NI	Low	Low	Moderate
3. De Vos 2015	Moderate	Moderate	Low	Low	NI	Low	Low	Moderate
4. Fang 2018	Moderate	Low	Low	Low	NI	Low	Low	Moderate
5. Fernando 2012	Moderate	Low	Low	Low	NI	Low	Low	Moderate
6. Ginström Ernstad 2016	Moderate	Low	Low	Low	NI	Low	High	High
7. Ginström Ernstad 2019	Moderate	Low	Low	Low	NI	Low	NI	High
8. Hatimaz 2017	High	High	Low	Low	NI	Low	Low	High
9. Hattori 2019	Low	Low	Low	Low	NI	Low	Low	Moderate
10. Huang 2020	Low	Low	Low	Low	NI	Low	Low	Low
11. Ishihara 2014	Moderate	Low	Low	Low	High	Low	Low	High
12. Kallen 2010	Moderate	Low	Low	Low	NI	Low	Low	High
13. Kalra 2012	Low	Low	Low	Low	High	Low	Low	High
14. Kato 2012	Low	Low	Low	Low	Low	Low	Low	Low
15. Kausche 2001	High	High	Low	Low	NI	Low	Low	High
16. Levi-Setti 2018	Low	Low	Low	Low	Low	Low	Low	Low
17. Li 2017	High	Moderate	Low	Low	NI	Low	Low	High
18. Long 2020	High	Low	Low	Low	Low	Low	Low	Moderate
19. Litzsky 2018	High	High/Unclear	Low	Low	High	Low	Low	High
20. Marconi 2019	Moderate	Low	Low	Low	Moderate	Low	Low	Moderate
21. Martin 2012	Moderate	Low	Low	Low	NI	Low	Low	Moderate
22. Maxwell 2014	Moderate	Low	Low	Low	NI	Low	Low	Moderate
23. Milki 2003	High	Low	Low	Low	NI	Low	Low	High
24. Oron 2015	Low	Low	Low	Low	Low	Low	Low	Low
25. Sazonova 2011	Moderate	Low	Low	Low	Low	Low	Low	Moderate
26. Schwarzler 2004	Moderate	Low	Low	Low	Low	Low	Low	Moderate
27. Shi 2019	Low	Low	Low	Low	Low	Low	Low	Low
28. Sotiroska 2015	Moderate	Low	Low	Low	NI	Low	Low	Moderate
29. Spangmose 2020	Low	Low	Low	Low	Low	Low	Low	Low
30. Wang 2010	Low	Low	Low	Low	NI	Low	Low	Low
31. Wikland 2010	Moderate	Low	Low	Low	NI	Low	Low	Moderate
32. Zhou 2018	Moderate	Low	Low	Low	NI	Low	Low	Moderate
33. Zhu 2018	Moderate	Low	Low	Low	NI	Low	Low	Moderate

Supplementary Appendix (Appendix S1). Studies with potentially duplicate populations after comparisons of study centers and study periods.

Studies with potentially duplicate populations (assessed according to study periods and study centers – these were never included in the same analysis; the most appropriate per analysis were used):

1. Shi 2019 (used in the majority of analyses), Li 2017 (used in the network meta-analysis on "gender", and in the "gender" subgroup analysis of singletons vs. non-singletons, as Shi et al. did not provide separate data on gender of singletons and infants from multiple pregnancies);
2. Zhu 2018, Huang 2020;
3. Ginström Ernstad 2019 (only the frozen cycles were used and only for the pairwise meta-analysis), Spangmose 2020 (only the fresh cycles were used and only for the pairwise meta-analysis), Ginström Ernstad 2016 (used only in the network meta-analysis, instead of the aforementioned two studies, as it provided data on all 4 interventions), Wikland 2010, Kallen 2010, Sazonova 2011.

Supplementary figure 1 (Figure S1). Pairwise meta-analysis forest plots showing risk ratios for preterm delivery (gestational age at delivery < 37weeks) (a), low birth weight (< 2500 g) (b), perinatal death (c), male gender (d) and healthy neonate (e) following blastocyst- vs cleavage-stage transfer. Regarding the congenital anomaly, preterm delivery, low birth weight, and perinatal death outcomes, a pooled effect on the left side of the x-axis would favor the blastocyst-stage transfer (i.e., the blastocyst-stage transfer reduces the risk of the investigated outcome), while a pooled effect on the right side of the x-axis would favor the cleavage-stage transfer (i.e., the blastocyst-stage transfer increases the risk of the investigated outcome). In case of the gender outcome, a pooled effect on the left side of the x-axis would indicate that cleavage-stage transfer increases the probability of a male liveborn infant compared to the blastocyst one, while a pooled effect on the right side of the x-axis would demonstrate an increase of that probability following blastocyst-stage transfer when compared to the cleavage-stage transfer. In case of the healthy neonate outcome, a pooled effect on the right side of the x-axis would favor the blastocyst-stage transfer (i.e., it increases the probability of a healthy neonate compared to cleavage-stage transfer). Abbreviations: 95% CI: 95% confidence interval; DL: DerSimonian and Laird random effects meta-analysis model; τ^2: tau squared estimate (estimated standard deviation of underlying effects across studies in the random effects meta-analysis); I^2: I-squared heterogeneity statistic.

a.

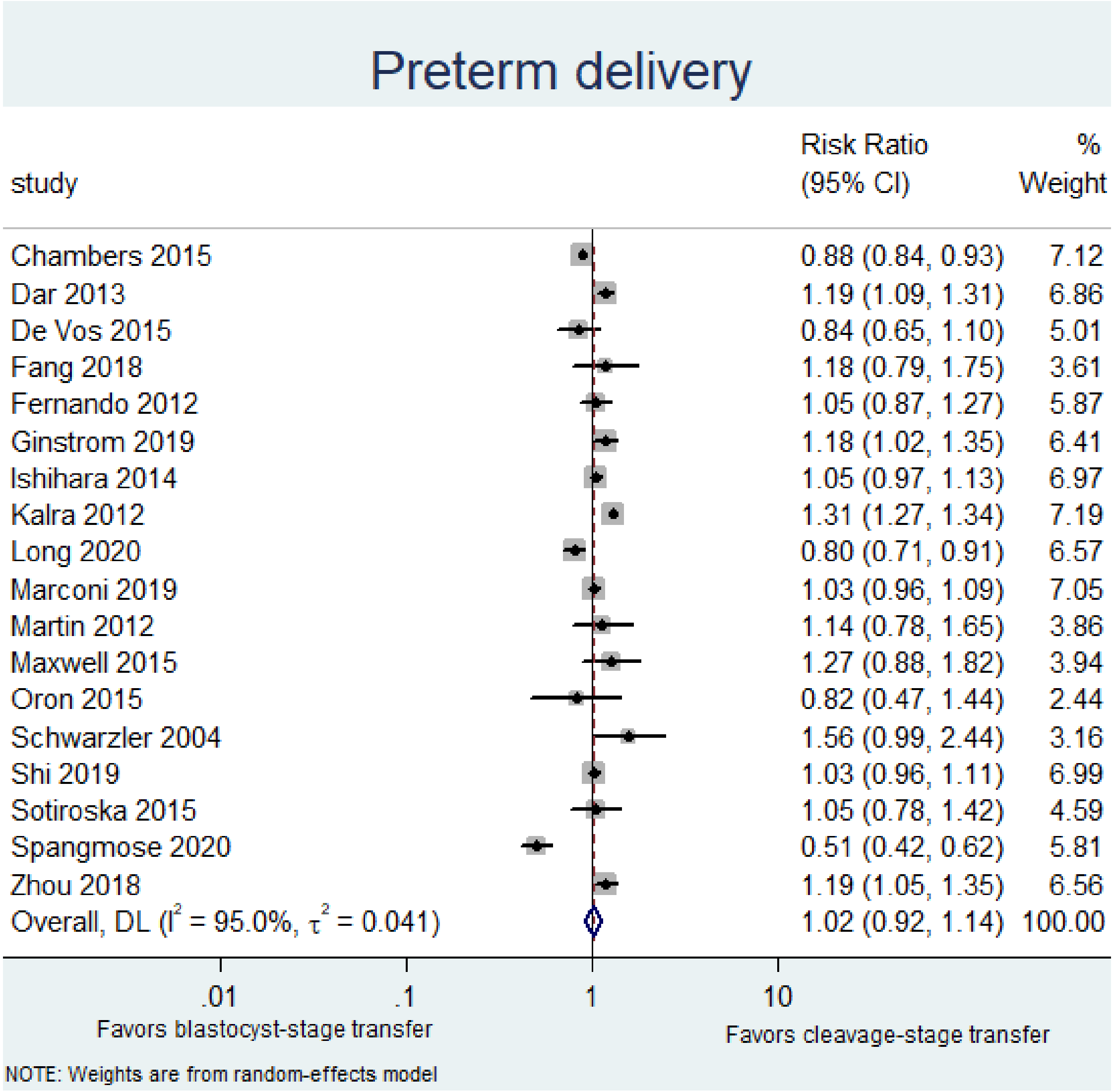

b.

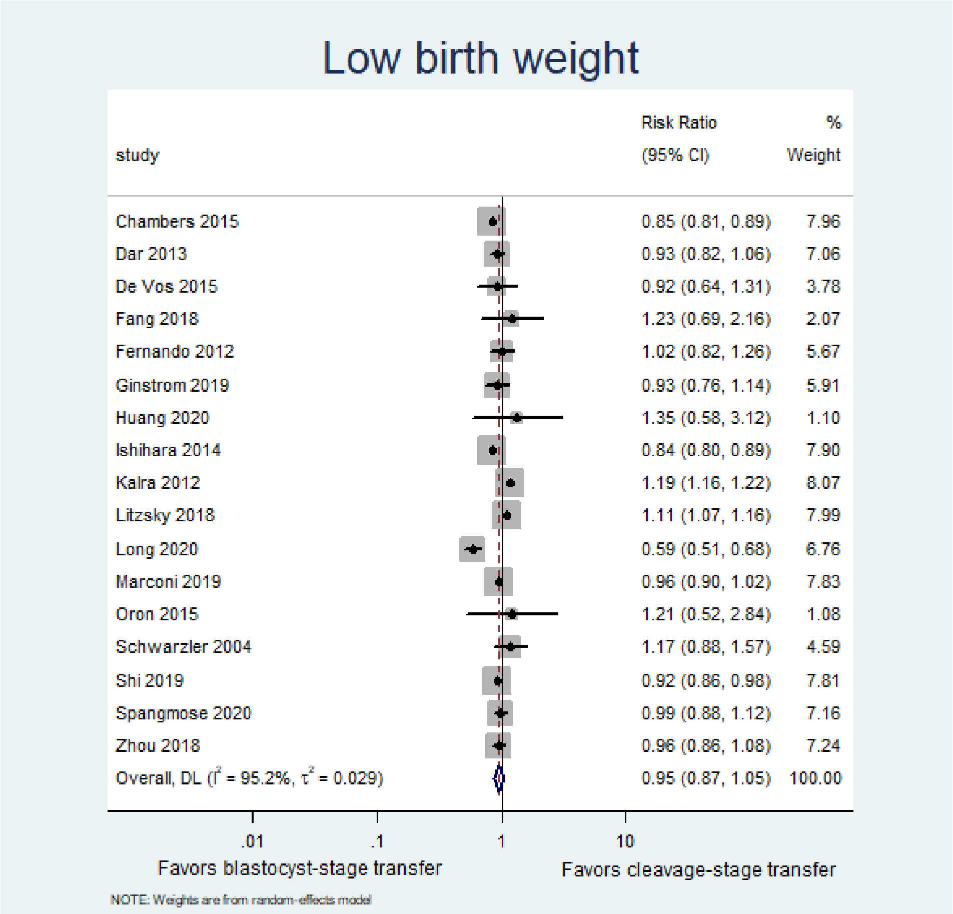
Low birth weight
study
Risk Ratio (95% CI)
% Weight
Chambers 2015 0.85 (0.81, 0.89) 7.96
Dar 2013 0.93 (0.82, 1.06) 7.06
De Vos 2015 0.92 (0.64, 1.31) 3.78
Fang 2018 1.23 (0.69, 2.16) 2.07
Fernando 2012 1.02 (0.82, 1.26) 5.67
Ginstrom 2019 0.93 (0.76, 1.14) 5.91
Huang 2020 1.35 (0.58, 3.12) 1.10
Ishihara 2014 0.84 (0.80, 0.89) 7.90
Kalra 2012 1.19 (1.16, 1.22) 8.07
Litzsky 2018 1.11 (1.07, 1.16) 7.99
Long 2020 0.59 (0.51, 0.68) 6.76
Marconi 2019 0.96 (0.90, 1.02) 7.83
Oron 2015 1.21 (0.52, 2.84) 1.08
Schwarzler 2004 1.17 (0.88, 1.57) 4.59
Shi 2019 0.92 (0.86, 0.98) 7.81
Spangmose 2020 0.99 (0.88, 1.12) 7.16
Zhou 2018 0.96 (0.86, 1.08) 7.24
Overall, DL (I² = 95.2%, τ² = 0.029) 0.95 (0.87, 1.05) 100.00
.01
.1
1
10
Favors blastocyst-stage transfer
Favors cleavage-stage transfer
NOTE: Weights are from random-effects model

c.

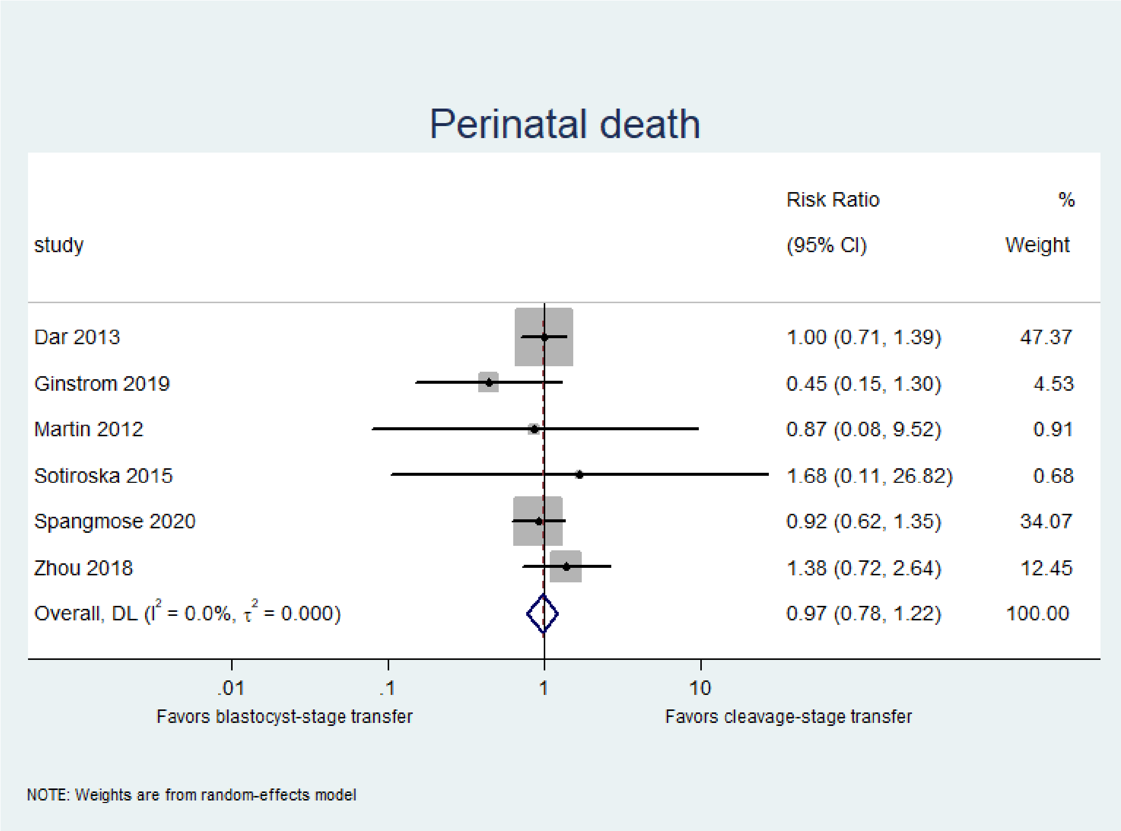
Perinatal death
study
Risk Ratio (95% CI)
% Weight
Dar 2013 1.00 (0.71, 1.39) 47.37
Ginstrom 2019 0.45 (0.15, 1.30) 4.53
Martin 2012 0.87 (0.08, 9.52) 0.91
Sotiroska 2015 1.68 (0.11, 26.82) 0.68
Spangmose 2020 0.92 (0.62, 1.35) 34.07
Zhou 2018 1.38 (0.72, 2.64) 12.45
Overall, DL (I² = 0.0%, τ² = 0.000) 0.97 (0.78, 1.22) 100.00
.01
.1
1
10
Favors blastocyst-stage transfer
Favors cleavage-stage transfer
NOTE: Weights are from random-effects model

d.

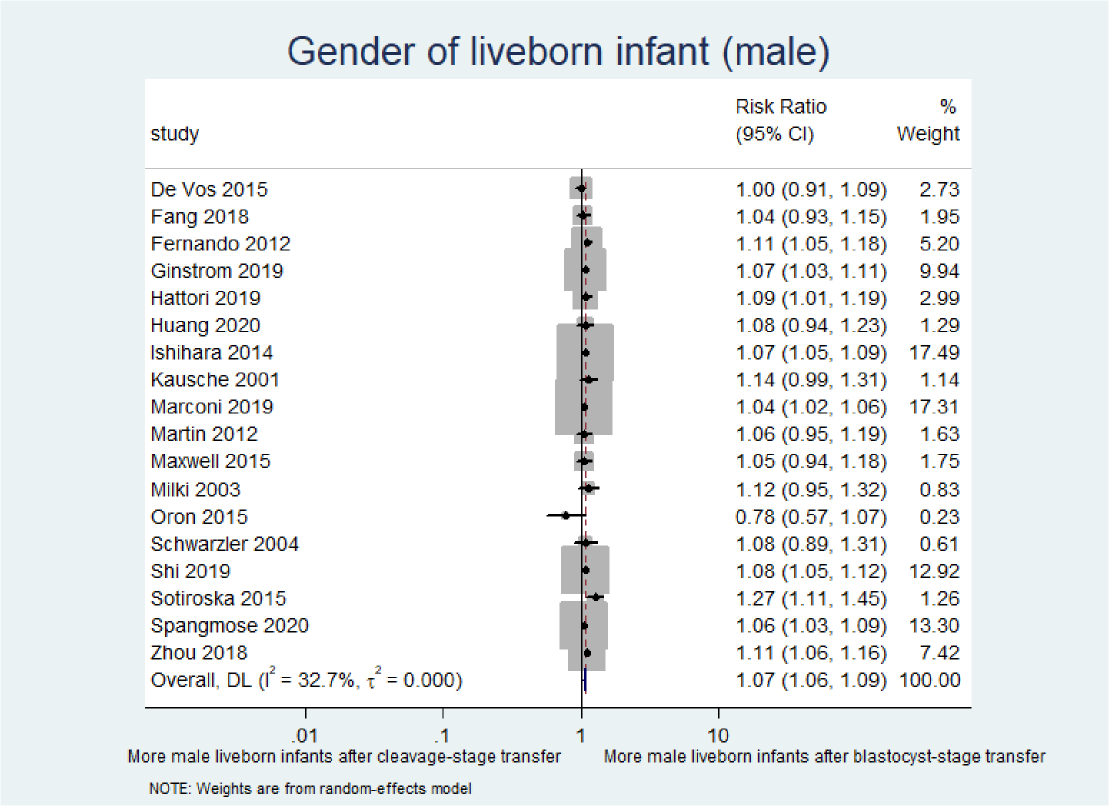
Gender of liveborn infant (male)
study
Risk Ratio (95% CI)
% Weight
De Vos 2015 1.00 (0.91, 1.09) 2.73
Fang 2018 1.04 (0.93, 1.15) 1.95
Fernando 2012 1.11 (1.05, 1.18) 5.20
Ginstrom 2019 1.07 (1.03, 1.11) 9.94
Hattori 2019 1.09 (1.01, 1.19) 2.99
Huang 2020 1.08 (0.94, 1.23) 1.29
Ishihara 2014 1.07 (1.05, 1.09) 17.49
Kausche 2001 1.14 (0.99, 1.31) 1.14
Marconi 2019 1.04 (1.02, 1.06) 17.31
Martin 2012 1.06 (0.95, 1.19) 1.63
Maxwell 2015 1.05 (0.94, 1.18) 1.75
Milki 2003 1.12 (0.95, 1.32) 0.83
Oron 2015 0.78 (0.57, 1.07) 0.23
Schwarzler 2004 1.08 (0.89, 1.31) 0.61
Shi 2019 1.08 (1.05, 1.12) 12.92
Sotiroska 2015 1.27 (1.11, 1.45) 1.26
Spangmose 2020 1.06 (1.03, 1.09) 13.30
Zhou 2018 1.11 (1.06, 1.16) 7.42
Overall, DL (I^2 = 32.7%, τ^2 = 0.000) 1.07 (1.06, 1.09) 100.00
.01
.1
1
10
More male liveborn infants after cleavage-stage transfer
More male liveborn infants after blastocyst-stage transfer
NOTE: Weights are from random-effects model

e.

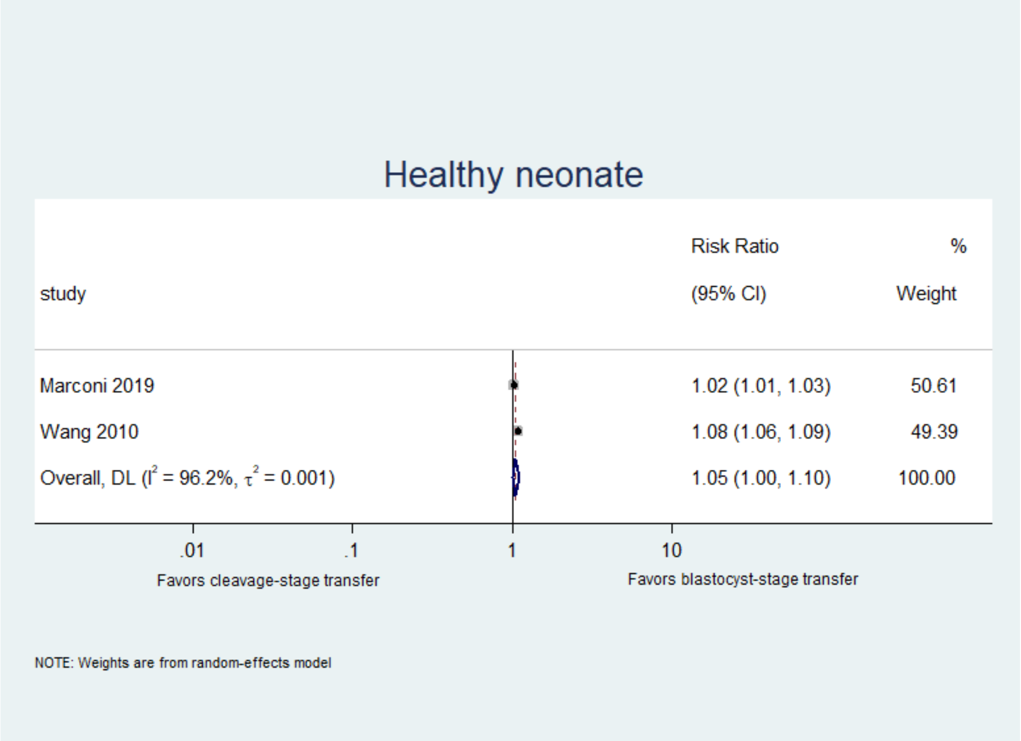
Healthy neonate
study
Risk Ratio (95% CI)
% Weight
Marconi 2019 1.02 (1.01, 1.03) 50.61
Wang 2010 1.08 (1.06, 1.09) 49.39
Overall, DL (I^2 = 96.2%, τ^2 = 0.001) 1.05 (1.00, 1.10) 100.00
.01
.1
1
10
Favors cleavage-stage transfer
Favors blastocyst-stage transfer
NOTE: Weights are from random-effects model

Supplementary figure 2 (Figure S2). Pairwise meta-analysis forest plots showing risk ratios in liveborn singleton and multiple pregnancies for any congenital anomaly (a), preterm delivery (gestational age at delivery < 37 weeks) (b), low birth weight (< 2500 g) (c), perinatal death (d) and male gender (e) following blastocyst vs cleavage transfer. Regarding the congenital anomaly, preterm delivery, low birth weight, and perinatal death outcomes, a pooled effect on the left side of the x-axis would favor the blastocyst-stage transfer (i.e., the blastocyst-stage transfer reduces the risk of the investigated outcome), while a pooled effect on the right side of the x-axis would favor the cleavage-stage transfer (i.e., the blastocyst-stage transfer increases the risk of the investigated outcome). In case of the gender outcome, a pooled effect on the left side of the x-axis would indicate that cleavage-stage transfer increases the probability of a male liveborn infant compared to the blastocyst one, while a pooled effect on the right side of the x-axis would demonstrate an increase of that probability following blastocyst-stage transfer in comparison with the cleavage-stage transfer. In case of the healthy neonate outcome, a pooled effect on the right side of the x-axis would favor the blastocyst-stage transfer (i.e., it increases the probability of a healthy neonate compared to cleavage-stage transfer). Abbreviations: 95% CI: 95% confidence interval; DL: DerSimonian and Laird random effects meta-analysis model; τ^2: tau squared estimate (estimated standard deviation of underlying effects across studies in the random effects meta-analysis); I^2: I-squared heterogeneity statistic.

a.

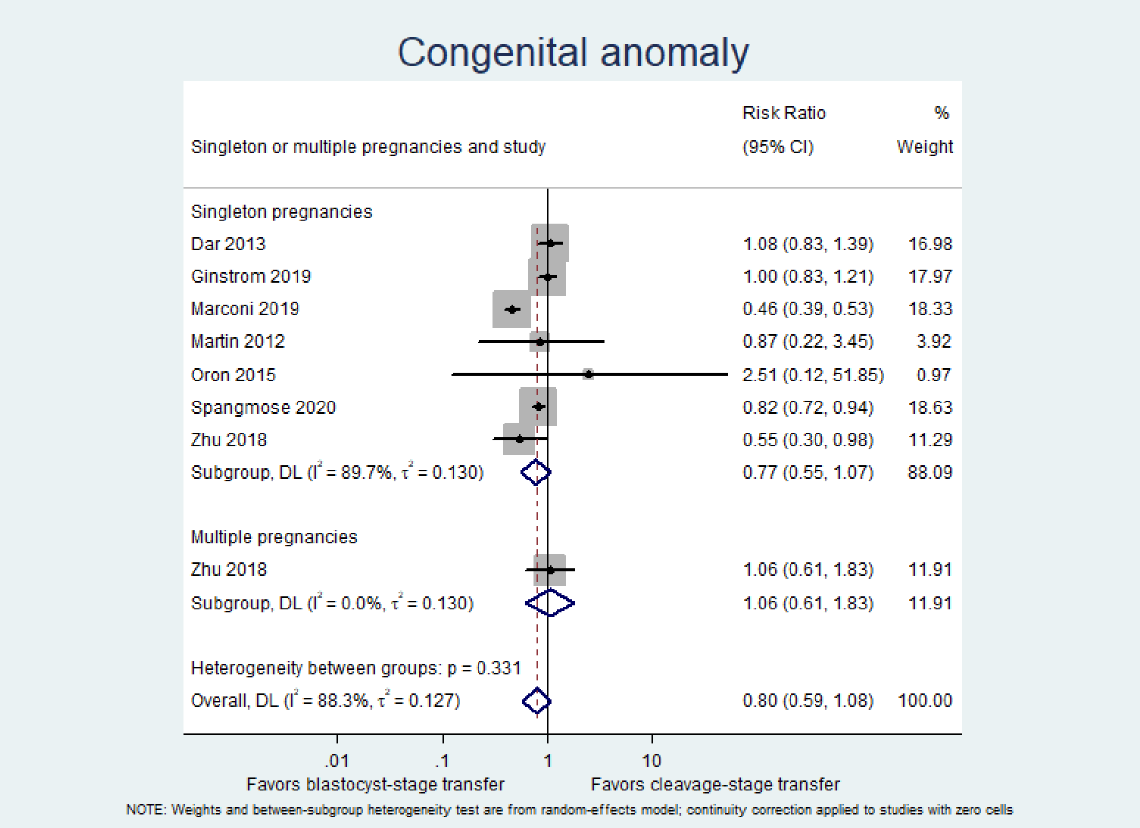

b.

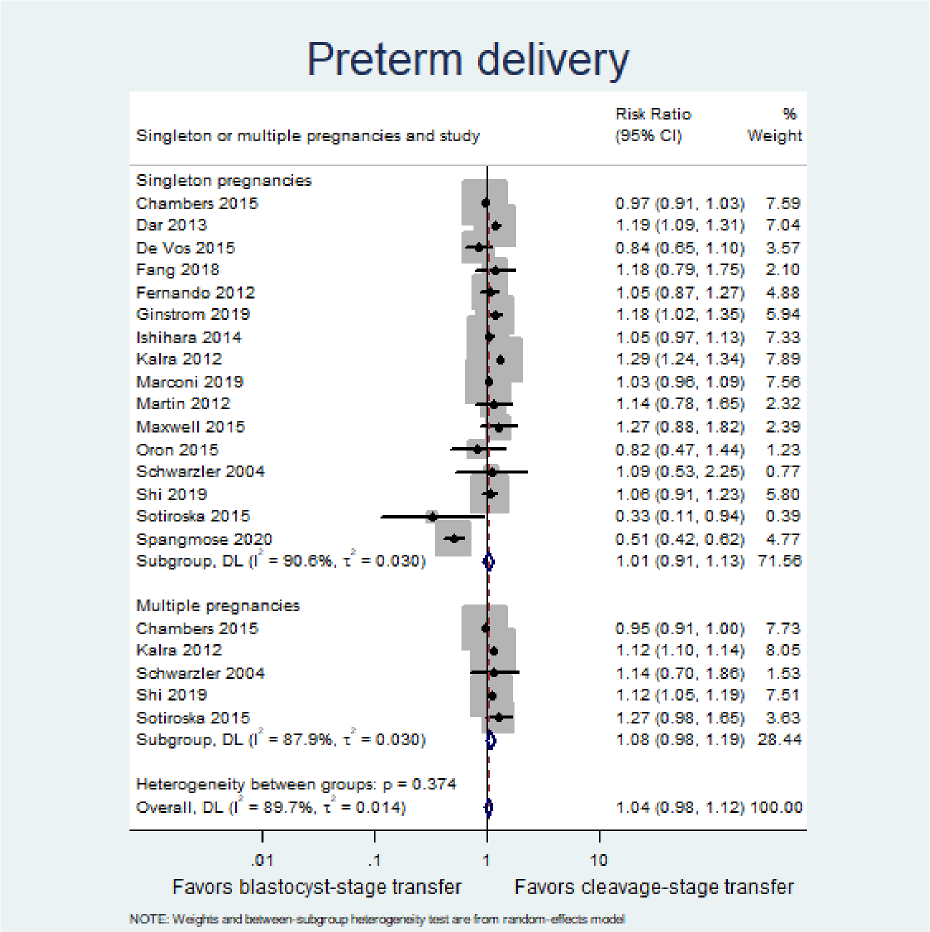
Preterm delivery
Singleton or multiple pregnancies and study
Risk Ratio (95% CI)
% Weight
Singleton pregnancies
Chambers 2015 0.97 (0.91, 1.03) 7.59
Dar 2013 1.19 (1.09, 1.31) 7.04
De Vos 2015 0.84 (0.65, 1.10) 3.57
Fang 2018 1.18 (0.79, 1.75) 2.10
Fernando 2012 1.05 (0.87, 1.27) 4.88
Ginstrom 2019 1.18 (1.02, 1.35) 5.94
Ishihara 2014 1.05 (0.97, 1.13) 7.33
Kalra 2012 1.29 (1.24, 1.34) 7.89
Marconi 2019 1.03 (0.96, 1.09) 7.56
Martin 2012 1.14 (0.78, 1.65) 2.32
Maxwell 2015 1.27 (0.88, 1.82) 2.39
Oron 2015 0.82 (0.47, 1.44) 1.23
Schwarzler 2004 1.09 (0.53, 2.25) 0.77
Shi 2019 1.06 (0.91, 1.23) 5.80
Sotiroska 2015 0.33 (0.11, 0.94) 0.39
Spangmose 2020 0.51 (0.42, 0.62) 4.77
Subgroup, DL (I² = 90.6%, τ² = 0.030) 1.01 (0.91, 1.13) 71.56
Multiple pregnancies
Chambers 2015 0.95 (0.91, 1.00) 7.73
Kalra 2012 1.12 (1.10, 1.14) 8.05
Schwarzler 2004 1.14 (0.70, 1.86) 1.53
Shi 2019 1.12 (1.05, 1.19) 7.51
Sotiroska 2015 1.27 (0.98, 1.65) 3.63
Subgroup, DL (I² = 87.9%, τ² = 0.030) 1.08 (0.98, 1.19) 28.44
Heterogeneity between groups: p = 0.374
Overall, DL (I² = 89.7%, τ² = 0.014) 1.04 (0.98, 1.12) 100.00
.01
.1
1
10
Favors blastocyst-stage transfer
Favors cleavage-stage transfer
NOTE: Weights and between-subgroup heterogeneity test are from random-effects model

c.

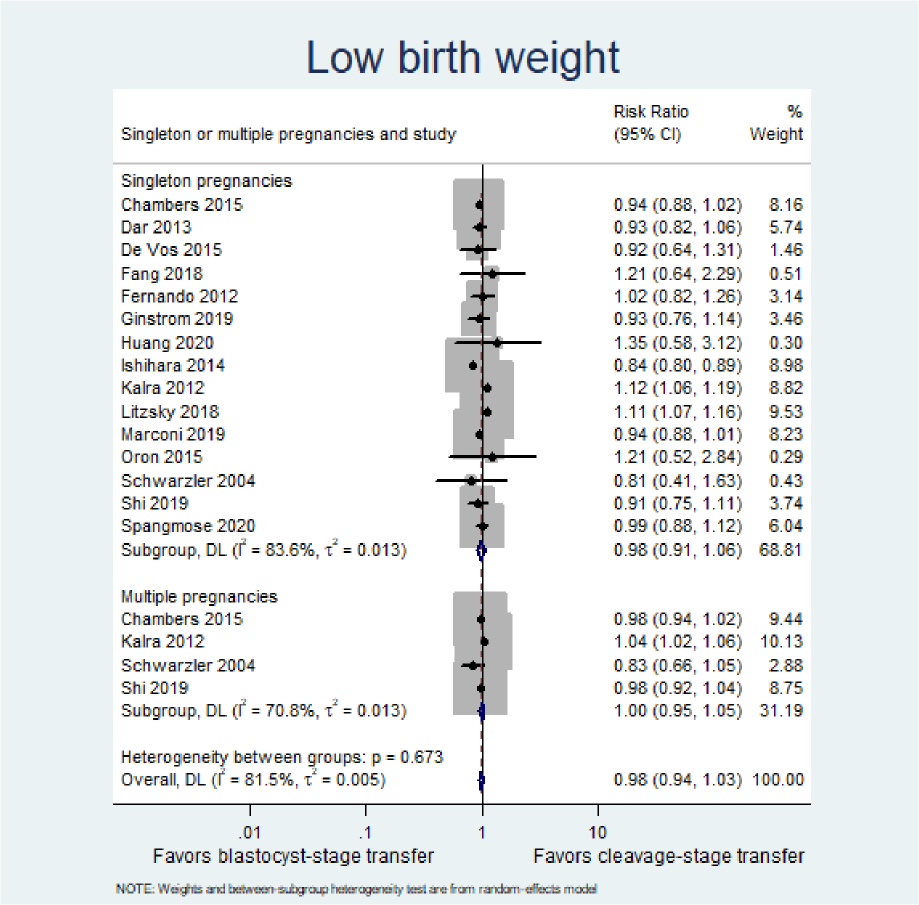
Low birth weight
Singleton or multiple pregnancies and study
Risk Ratio (95% CI)
% Weight
Singleton pregnancies
Chambers 2015 0.94 (0.88, 1.02) 8.16
Dar 2013 0.93 (0.82, 1.06) 5.74
De Vos 2015 0.92 (0.64, 1.31) 1.46
Fang 2018 1.21 (0.64, 2.29) 0.51
Fernando 2012 1.02 (0.82, 1.26) 3.14
Ginstrom 2019 0.93 (0.76, 1.14) 3.46
Huang 2020 1.35 (0.58, 3.12) 0.30
Ishihara 2014 0.84 (0.80, 0.89) 8.98
Kalra 2012 1.12 (1.06, 1.19) 8.82
Litzsky 2018 1.11 (1.07, 1.16) 9.53
Marconi 2019 0.94 (0.88, 1.01) 8.23
Oron 2015 1.21 (0.52, 2.84) 0.29
Schwarzler 2004 0.81 (0.41, 1.63) 0.43
Shi 2019 0.91 (0.75, 1.11) 3.74
Spangmose 2020 0.99 (0.88, 1.12) 6.04
Subgroup, DL (I² = 83.6%, τ² = 0.013) 0.98 (0.91, 1.06) 68.81
Multiple pregnancies
Chambers 2015 0.98 (0.94, 1.02) 9.44
Kalra 2012 1.04 (1.02, 1.06) 10.13
Schwarzler 2004 0.83 (0.66, 1.05) 2.88
Shi 2019 0.98 (0.92, 1.04) 8.75
Subgroup, DL (I² = 70.8%, τ² = 0.013) 1.00 (0.95, 1.05) 31.19
Heterogeneity between groups: p = 0.673
Overall, DL (I² = 81.5%, τ² = 0.005) 0.98 (0.94, 1.03) 100.00
.01
.1
1
10
Favors blastocyst-stage transfer
Favors cleavage-stage transfer
NOTE: Weights and between-subgroup heterogeneity test are from random-effects model

d.

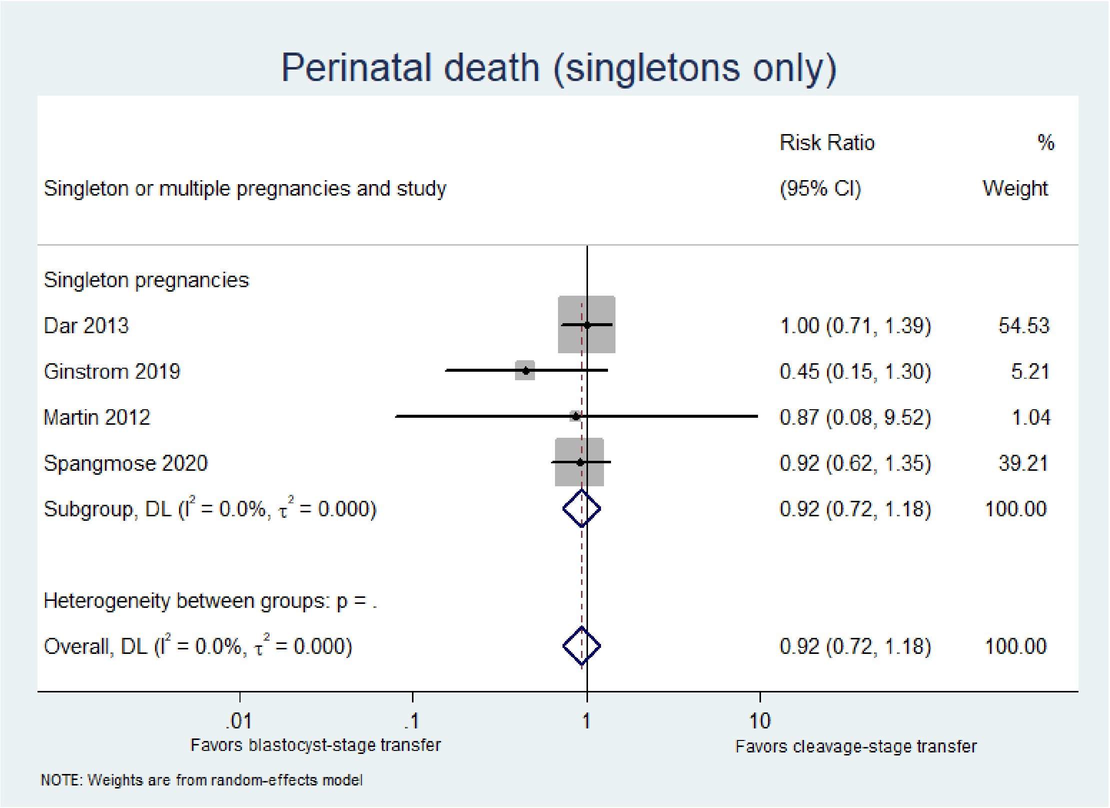

Perinatal death (singletons only)
Risk Ratio
(95% CI)
%
Weight
Singleton or multiple pregnancies and study
Singleton pregnancies
Dar 2013
1.00 (0.71, 1.39)
54.53
Ginstrom 2019
0.45 (0.15, 1.30)
5.21
Martin 2012
0.87 (0.08, 9.52)
1.04
Spangmose 2020
0.92 (0.62, 1.35)
39.21
Subgroup, DL (I^2 = 0.0%, τ^2 = 0.000)
0.92 (0.72, 1.18)
100.00
Heterogeneity between groups: p = .
Overall, DL (I^2 = 0.0%, τ^2 = 0.000)
0.92 (0.72, 1.18)
100.00
.01
.1
1
10
Favors blastocyst-stage transfer
Favors cleavage-stage transfer
NOTE: Weights are from random-effects model

e.

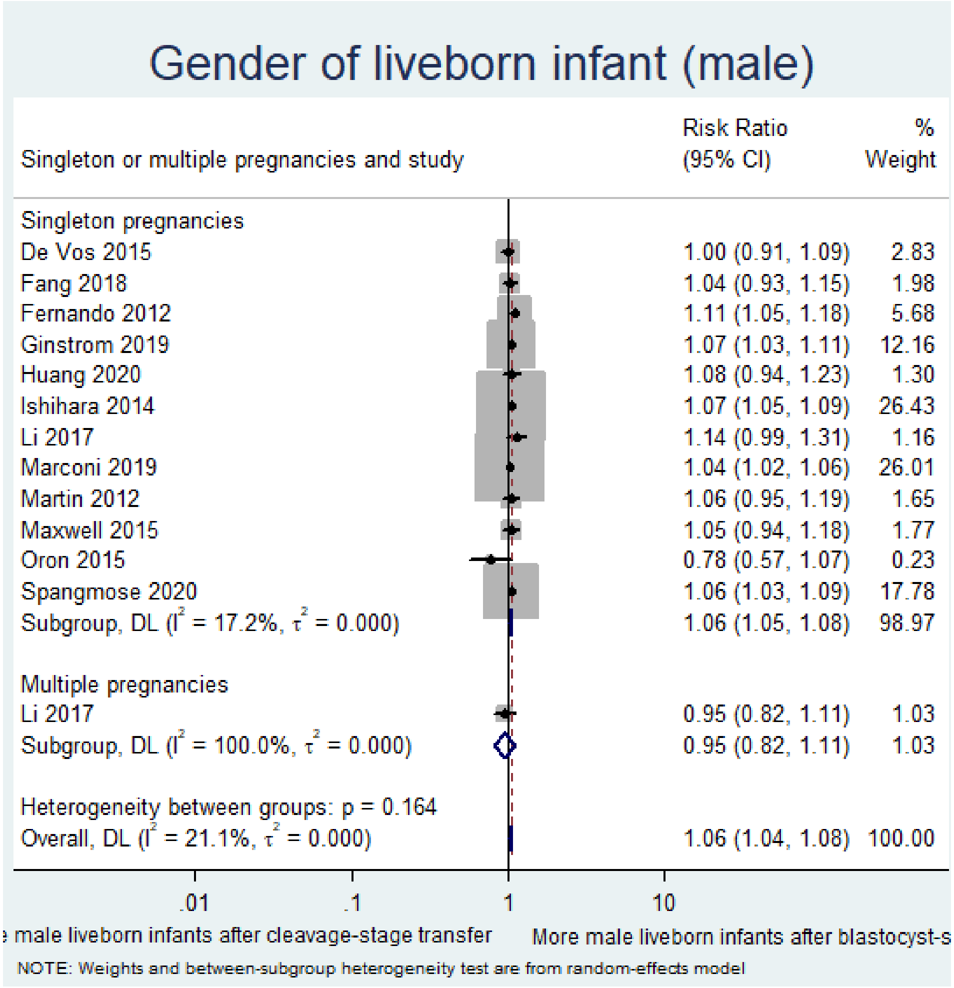

Gender of liveborn infant (male)
Singleton or multiple pregnancies and study
Risk Ratio
(95% CI)
%
Weight
Singleton pregnancies
De Vos 2015
1.00 (0.91, 1.09)
2.83
Fang 2018
1.04 (0.93, 1.15)
1.98
Fernando 2012
1.11 (1.05, 1.18)
5.68
Ginstrom 2019
1.07 (1.03, 1.11)
12.16
Huang 2020
1.08 (0.94, 1.23)
1.30
Ishihara 2014
1.07 (1.05, 1.09)
26.43
Li 2017
1.14 (0.99, 1.31)
1.16
Marconi 2019
1.04 (1.02, 1.06)
26.01
Martin 2012
1.06 (0.95, 1.19)
1.65
Maxwell 2015
1.05 (0.94, 1.18)
1.77
Oron 2015
0.78 (0.57, 1.07)
0.23
Spangmose 2020
1.06 (1.03, 1.09)
17.78
Subgroup, DL (I^2 = 17.2%, τ^2 = 0.000)
1.06 (1.05, 1.08)
98.97
Multiple pregnancies
Li 2017
0.95 (0.82, 1.11)
1.03
Subgroup, DL (I^2 = 100.0%, τ^2 = 0.000)
0.95 (0.82, 1.11)
1.03
Heterogeneity between groups: p = 0.164
Overall, DL (I^2 = 21.1%, τ^2 = 0.000)
1.06 (1.04, 1.08)
100.00
.01
.1
1
10
male liveborn infants after cleavage-stage transfer
More male liveborn infants after blastocyst-s
NOTE: Weights and between-subgroup heterogeneity test are from random-effects model

Supplementary figure 3 (Figure S3). Network geometry. The size of the nodes representing the four embryo transfer arms is indicative of the number of included studies per arm, while the thickness of the lines is indicative of the amount of data. Nodes representing studies on frozen cycles are smaller.

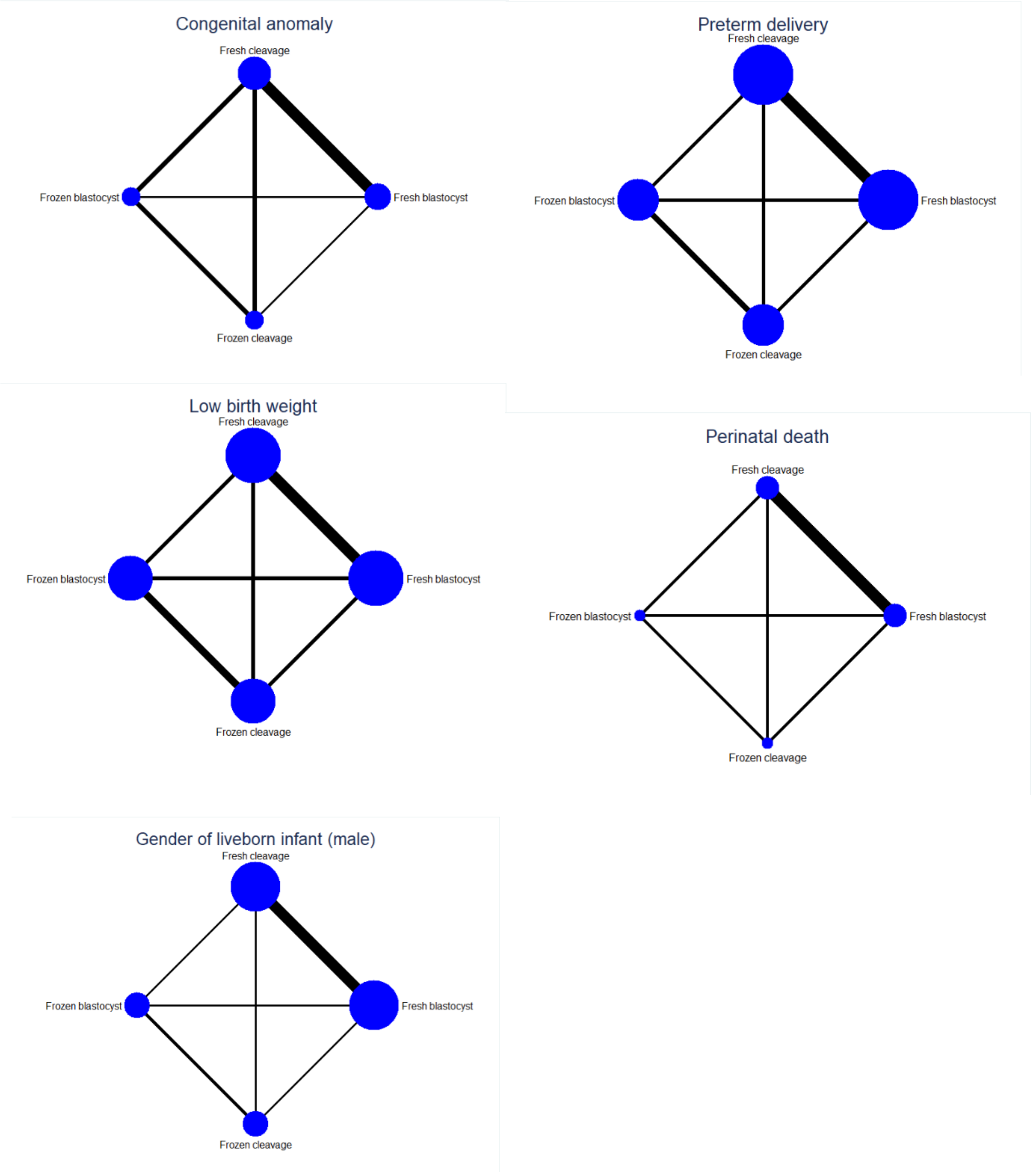

Supplementary figure 4 (Figure S4). Network sidesplitting of nodes, examining differences between direct and indirect evidence (measure of effect: risk ratio, log scale) within each comparison set (local tests on inconsistency). A symmetrical alternative of the Dias et al. method was employed.

a. Congenital anomaly

Side	Direct		Indirect		Difference		
	Coef.	Std. Err.	Coef.	Std. Err.	Coef.	Std. Err.	P>|z|
A B *	.4107253	.1976103	-.4453534	.9589597	.8560787	.9762175	0.381
A C	-.0120465	.3320612	.5844094	.4428779	-.5964559	.5532811	0.281
A D	.1138918	.1742747	1.144255	.3031428	-1.030363	.3454033	0.003
B C *	-.0929061	.1603879	-1.449561	.4261772	1.356655	.4095564	0.001
B D *	.1119703	.1431691	-1.244726	.4239714	1.356697	.4068661	0.001
C D	.	.	.	.	.	.	.

b. Preterm delivery

Side	Direct		Indirect		Difference		
	Coef.	Std. Err.	Coef.	Std. Err.	Coef.	Std. Err.	P>|z|
A B *	-.0826948	.0764439	472.477	2.396164	-472.5597	2.397507	0.000
A C *	.0539144	.1152161	-.2768414	.2008807	.3307558	.231843	0.154
A D *	-.0421656	.1255568	-.1177735	.2176066	.0756079	.2514033	0.764
B C *	.0362858	.1224299	.1118938	.2203375	-.0756079	.2514017	0.764
B D *	-.054018	.1117716	.2767378	.204212	-.3307558	.2318422	0.154
C D	.	.	.	.	.	.	.

c. Low birth weight

Side	Direct		Indirect		Difference		
	Coef.	Std. Err.	Coef.	Std. Err.	Coef.	Std. Err.	P>|z|
A B *	.0181457	.0793629	259.9735	18.87703	-259.9554	18.87722	0.000
A C *	-.2094708	.1281465	-.4826383	.2195287	.2731675	.255459	0.285
A D *	-.1015997	.1357385	-.1928331	.2409955	.0912333	.2768049	0.742
B C *	-.320522	.1367277	-.2292826	.2408566	-.0912393	.2768161	0.742
B D *	-.1999456	.1202955	.073221	.2266239	-.2731666	.255416	0.285
C D	.	.	.	.	.	.	.

d. Perinatal death

Side	Direct		Indirect		Difference		
	Coef.	Std. Err.	Coef.	Std. Err.	Coef.	Std. Err.	P>|z|
A B	.	.	.	.	.	.	.
A C *	.3562973	.2881456	1.277613	.4806096	-.9213155	.5481624	0.093
A D *	-.2293756	.2497092	.6919399	.4585983	-.9213155	.5481623	0.093
B C *	.8172237	.2327917	-.1040918	.5634654	.9213155	.5481623	0.093
B D *	.2315508	.1830814	-.6897647	.544812	.9213155	.5481624	0.093
C D	.	.	.	.	.	.	.

e. Gender (male)

Side	Direct		Indirect		Difference		
	Coef.	Std. Err.	Coef.	Std. Err.	Coef.	Std. Err.	P>|z|
A B *	-.046452	.0075645	-.0473458	125.2517	.0008938	125.2517	1.000
A C *	.0199863	.011562	.0268061	.0247457	-.0068198	.0289763	0.814
A D *	-.0459478	.0142865	-.0403891	.0250854	-.0055587	.0291296	0.849
B C *	.0683098	.0093407	.0627511	.0274127	.0055587	.0291296	0.849
B D *	.002276	.0114216	-.0045438	.0287478	.0068198	.0289762	0.814
C D	.	.	.	.	.	.	.

Supplementary figure 5 (Figure S5). Network meta-analysis forest plots showing risk ratio for any congenital anomaly (a), preterm delivery (gestational age at delivery < 37 weeks) (b), low birth weight (< 2500 g) (c), perinatal death (d) and male gender (e) following fresh-cleavage, fresh-blastocyst, frozen-cleavage or frozen-blastocyst transfer. Blue diamonds indicate the effect pooled within a certain design (i.e., comparison), while red diamonds demonstrate the effect estimate pooled within all designs. When considering an “Intervention 1” vs “Intervention 2” comparison (where interventions represent any 2 of the investigated modalities), a pooled effect on the right side of the x-axis would indicate that intervention 1 increases the risk for the examined outcome compared to intervention 2, while a pooled effect on the left side of the x-axis would indicate that intervention 1 decreases that risk compared to intervention 2.

a. Congenital anomaly

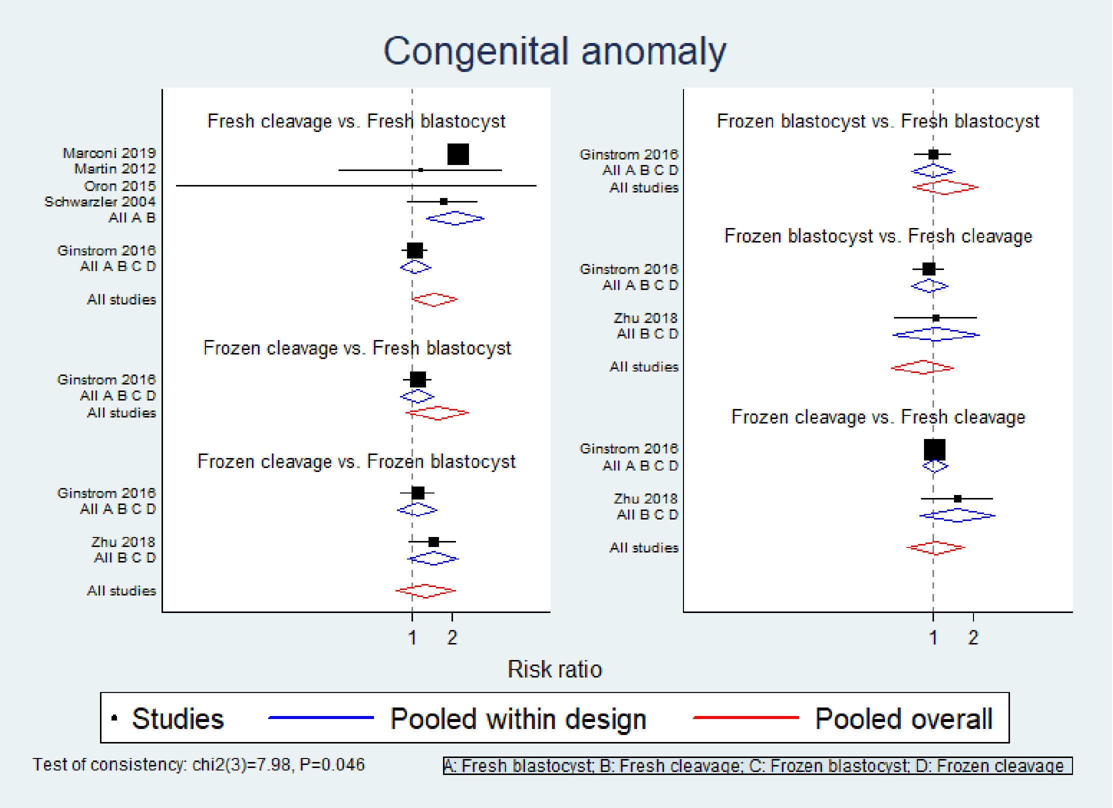

b. Preterm delivery

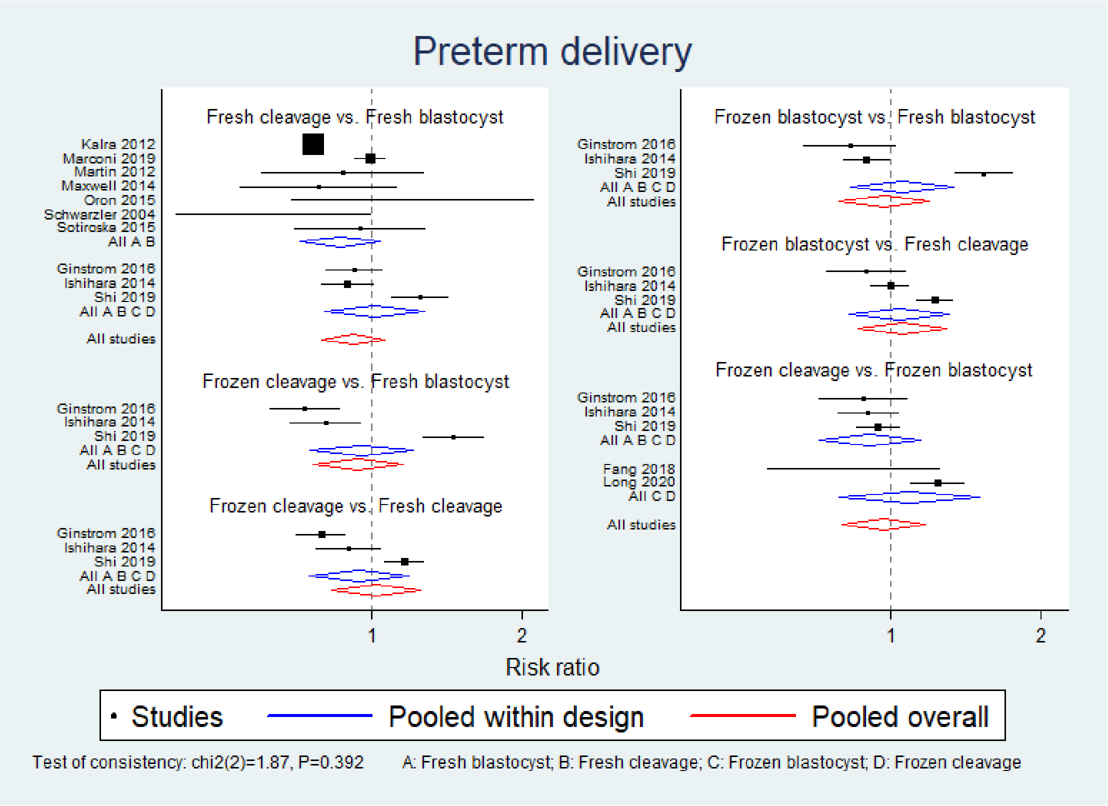

c. Low birth weight

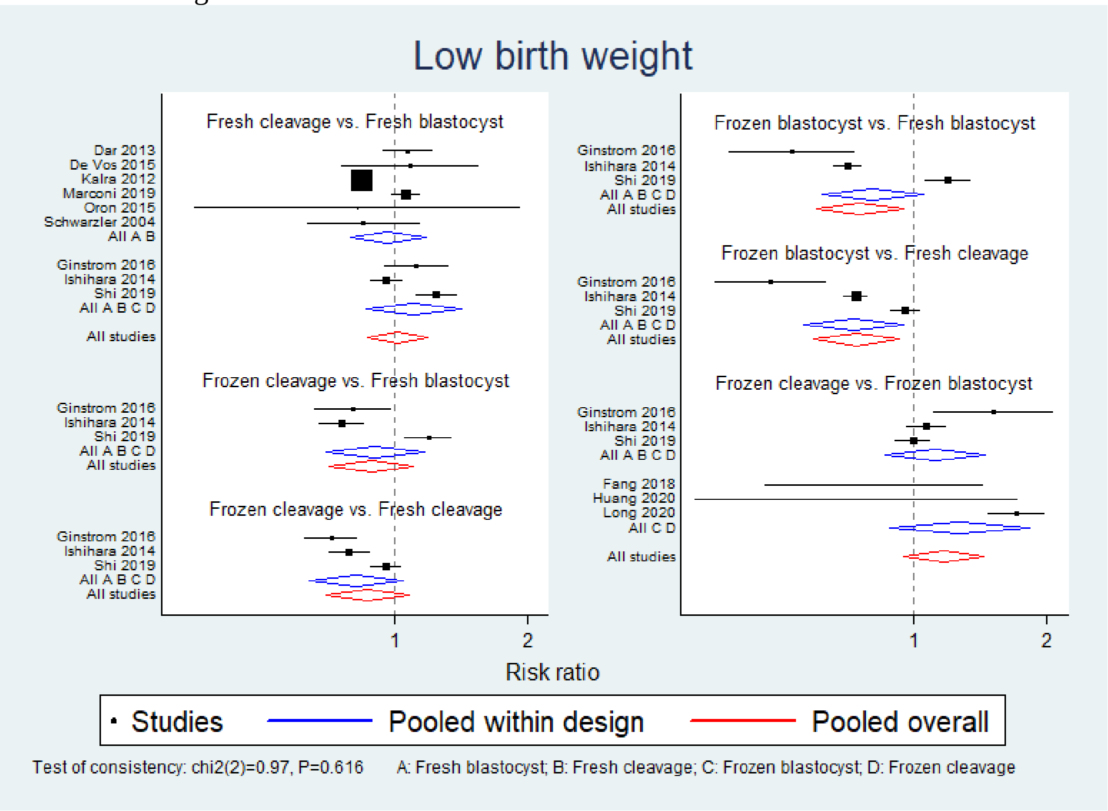

d. Perinatal death

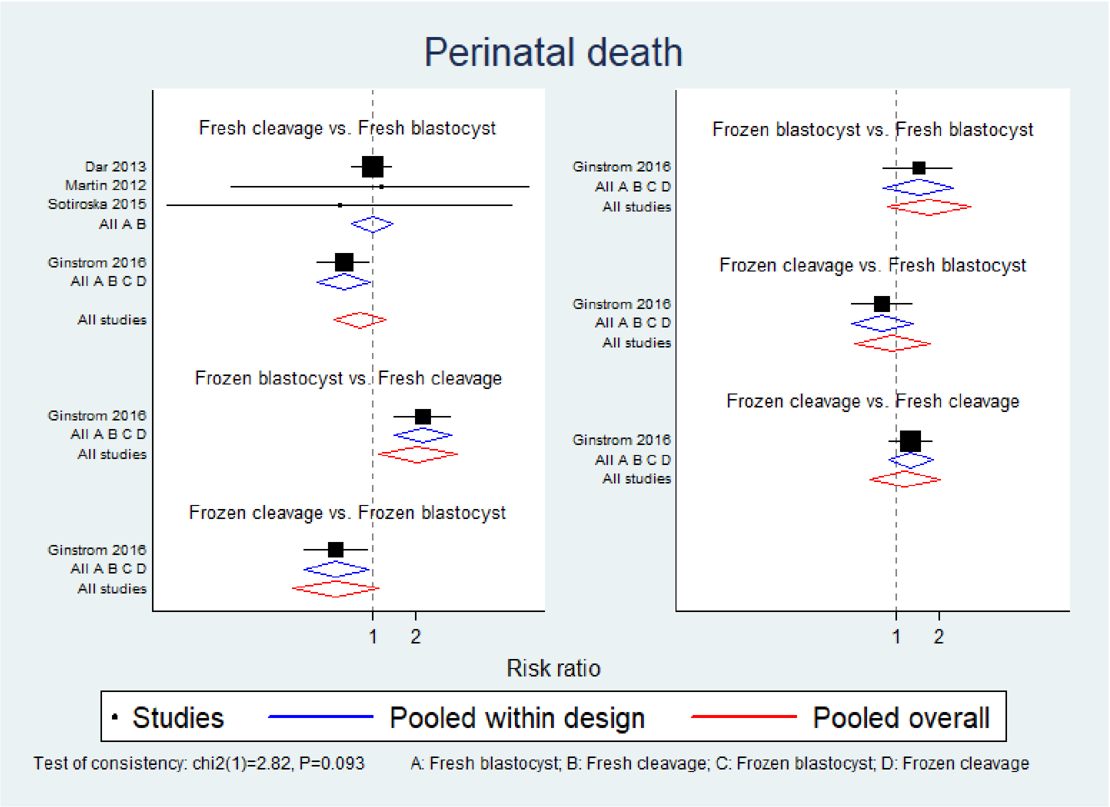

e. Gender of the liveborn infant (male)

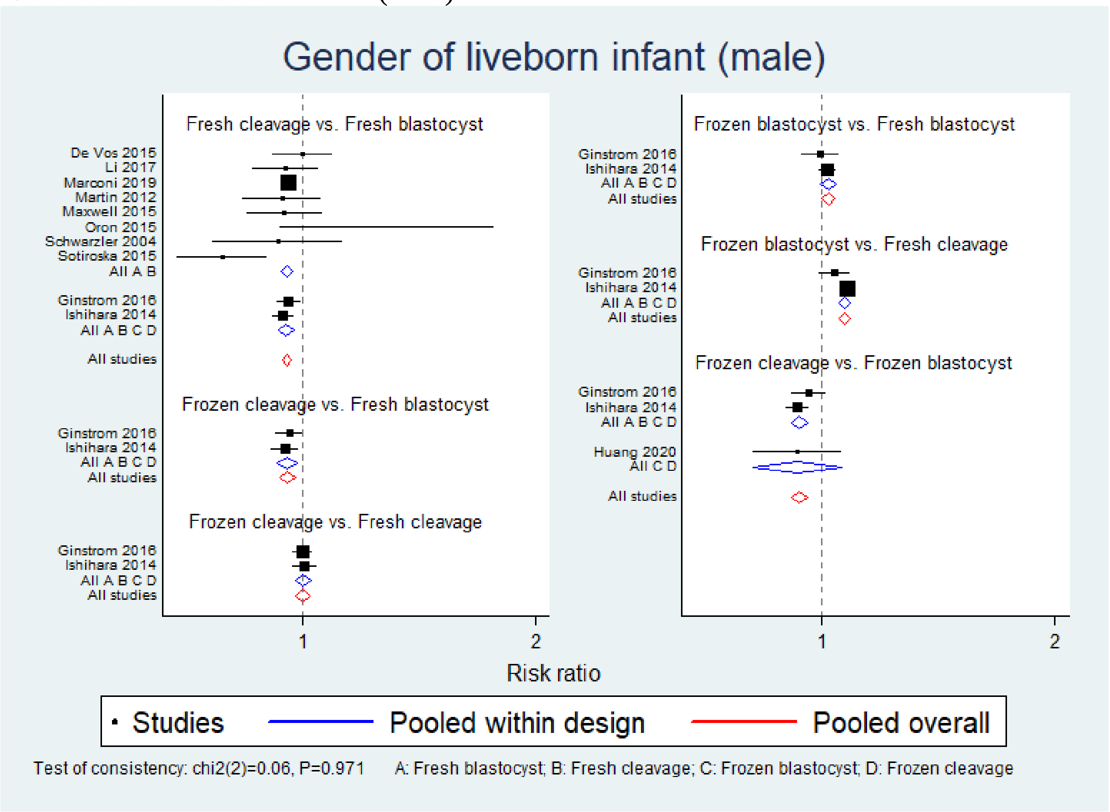

Supplementary figure 6 (Figure S6). Network meta-analyses for singleton pregnancies. Forest and interval plots for each outcome are presented. When considering an "Intervention 1" vs "Intervention 2" comparison (where interventions represent any 2 of the investigated modalities), a pooled effect on the right side of the x-axis would indicate that intervention 1 increases the risk for the examined outcome compared to intervention 2, while a pooled effect on the left side of the x-axis would indicate that intervention 1 decreases that risk compared to intervention 2. cleav, cleavage; blast, blastocyst.

a. Congenital anomaly

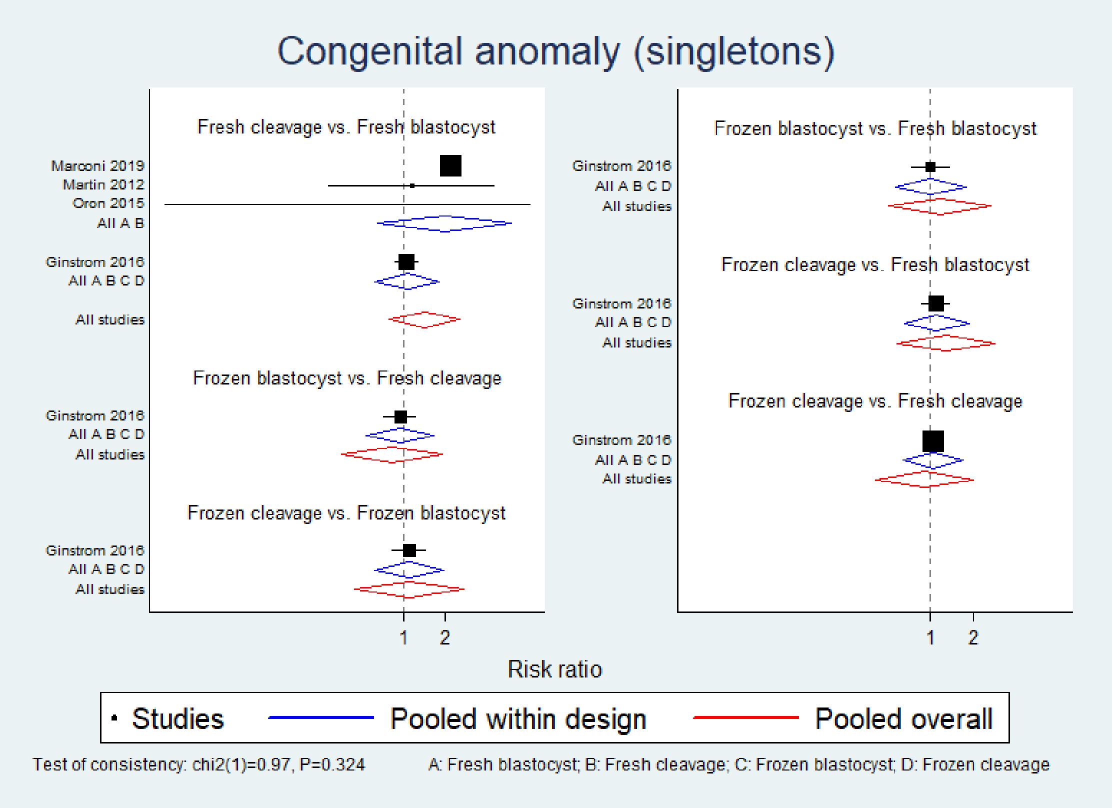

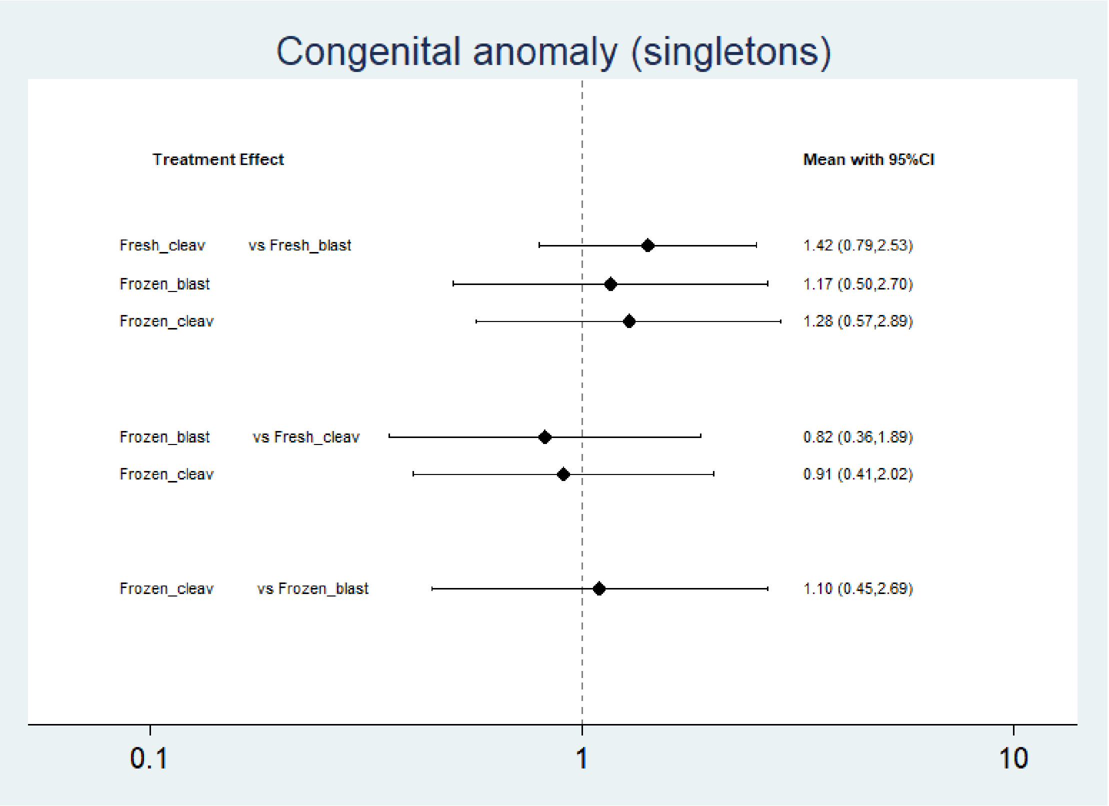

b. Preterm delivery

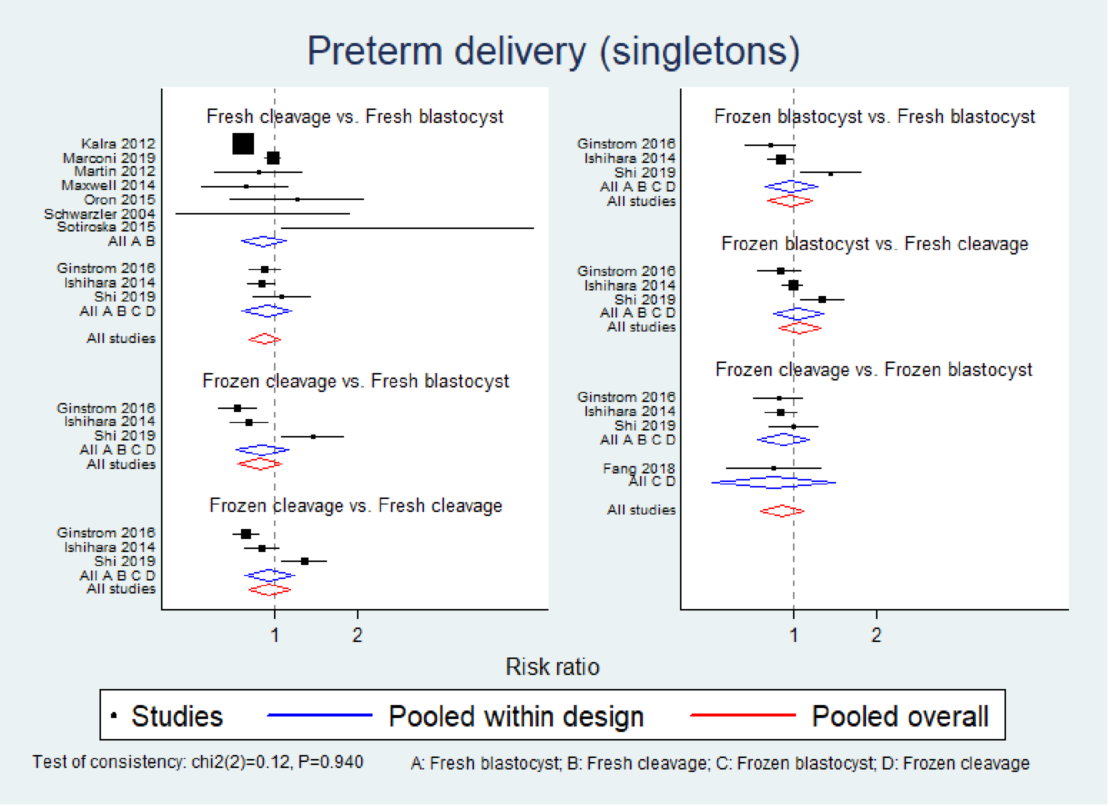

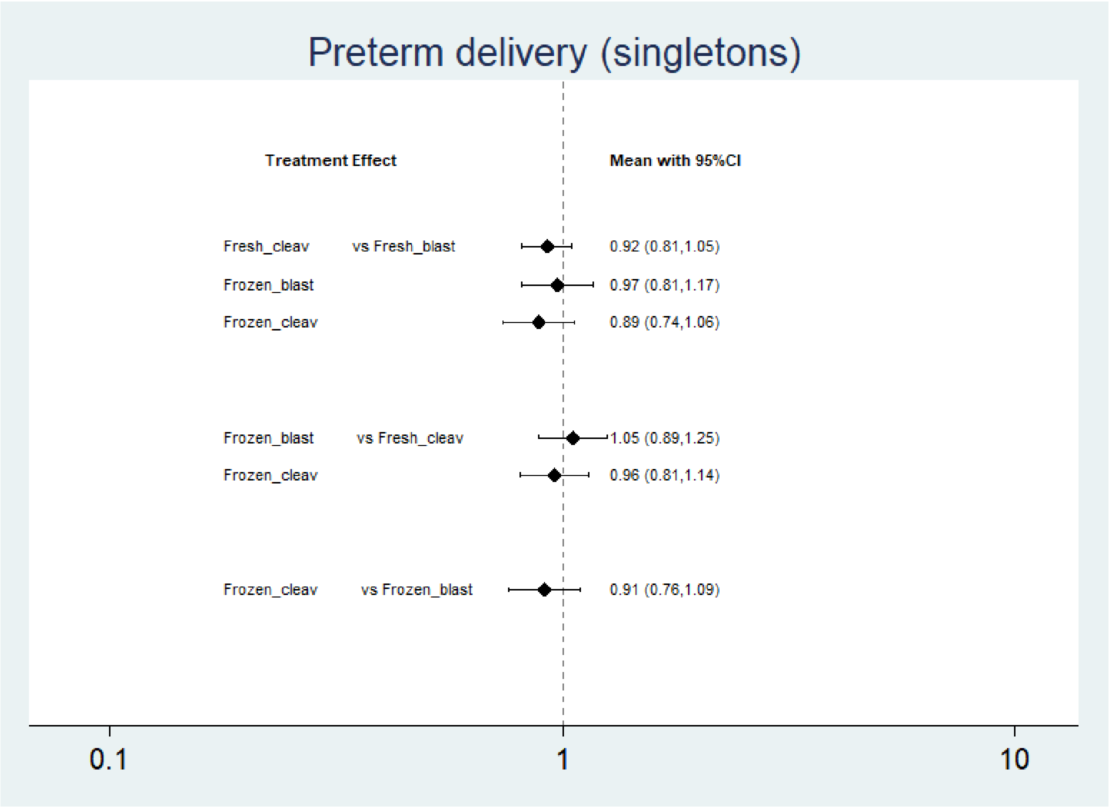

c. Low birth weight

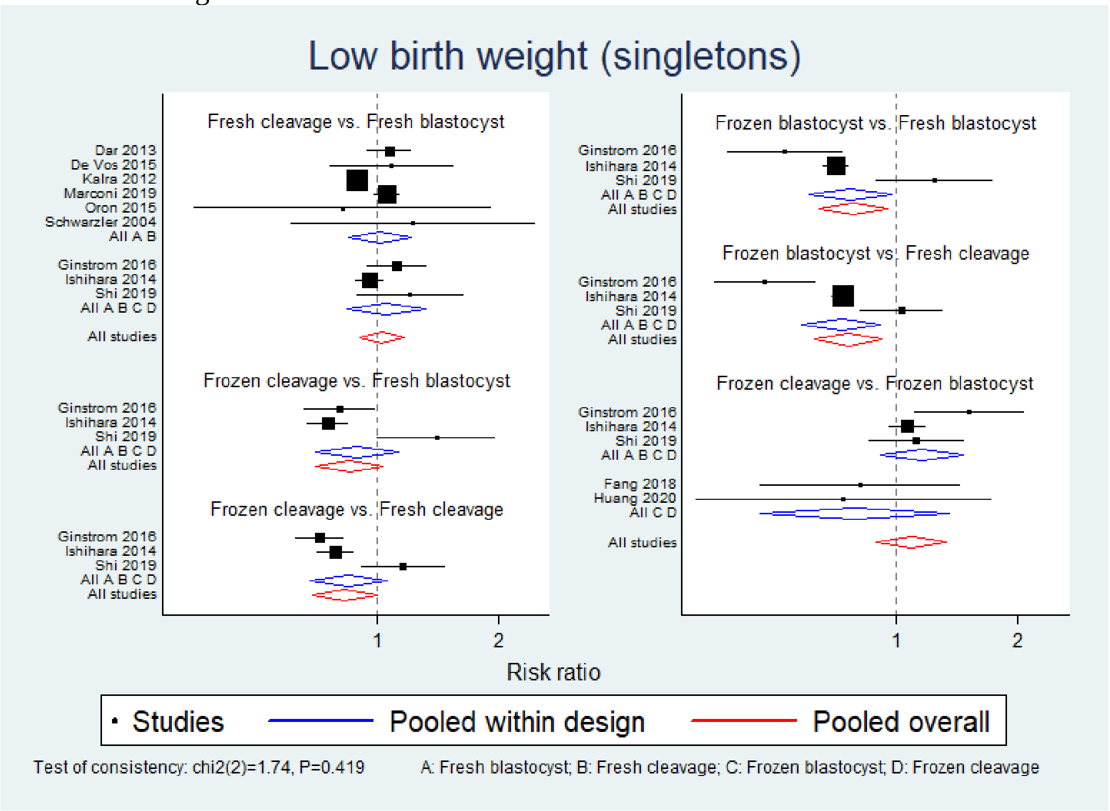

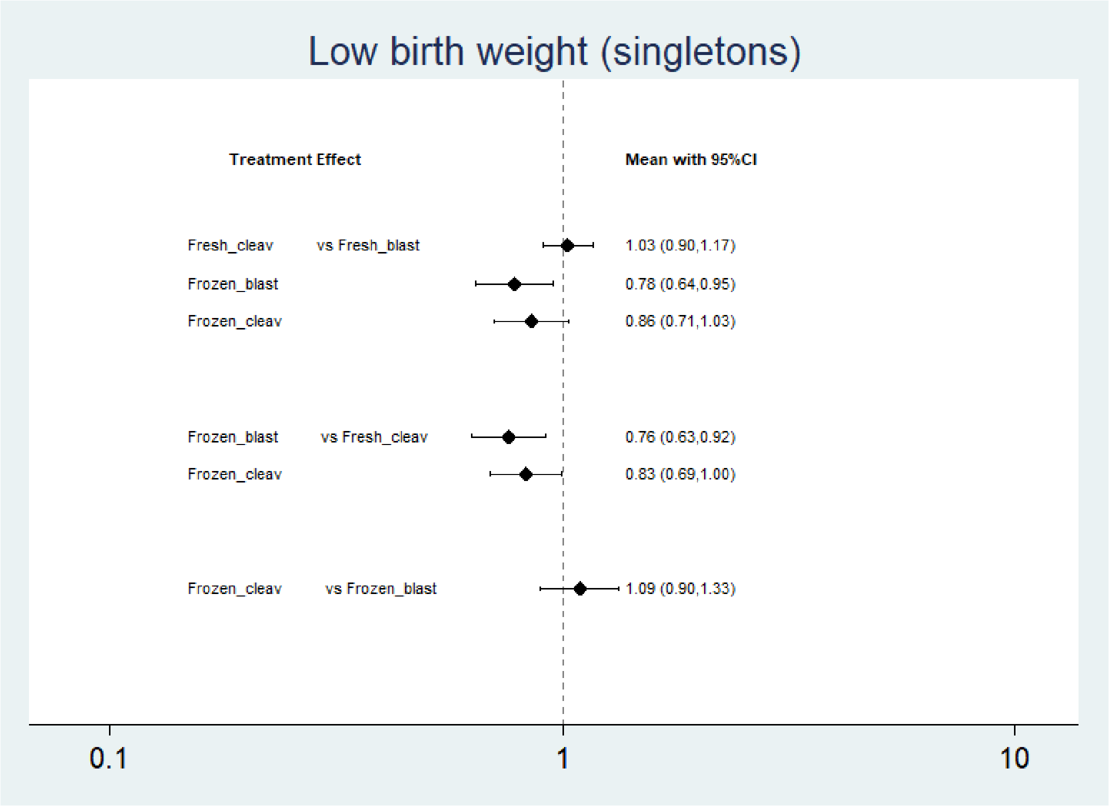

d. Perinatal death

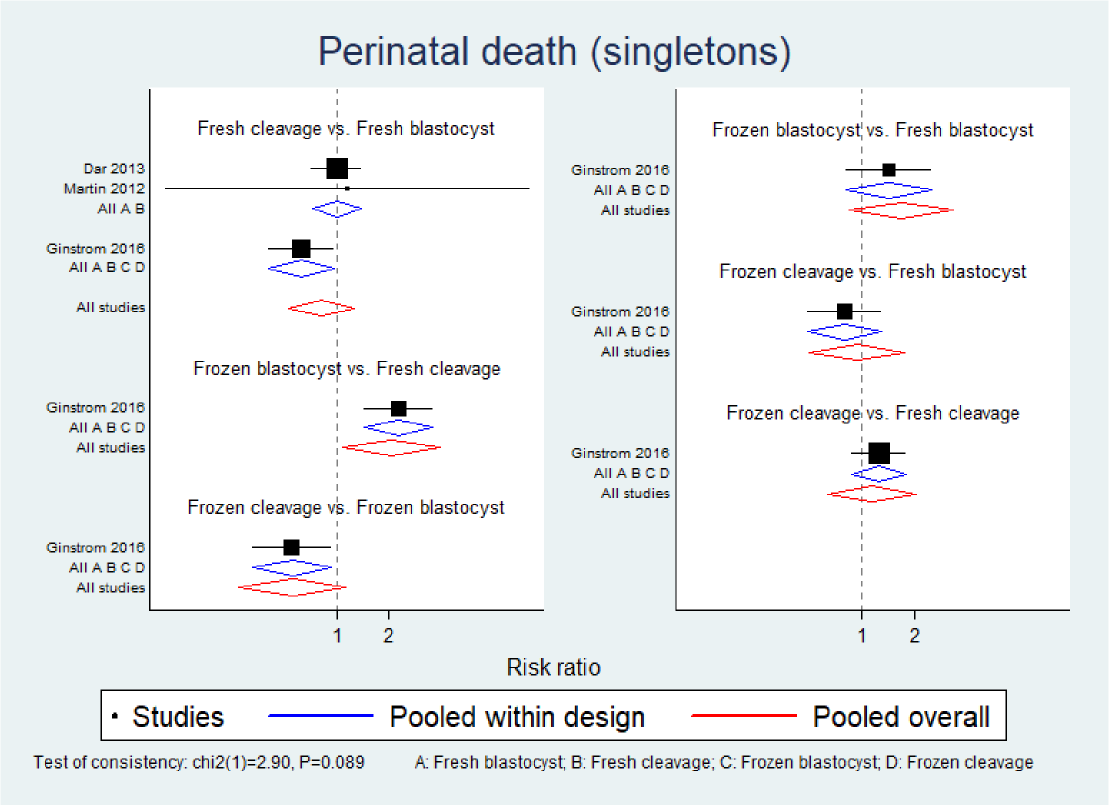

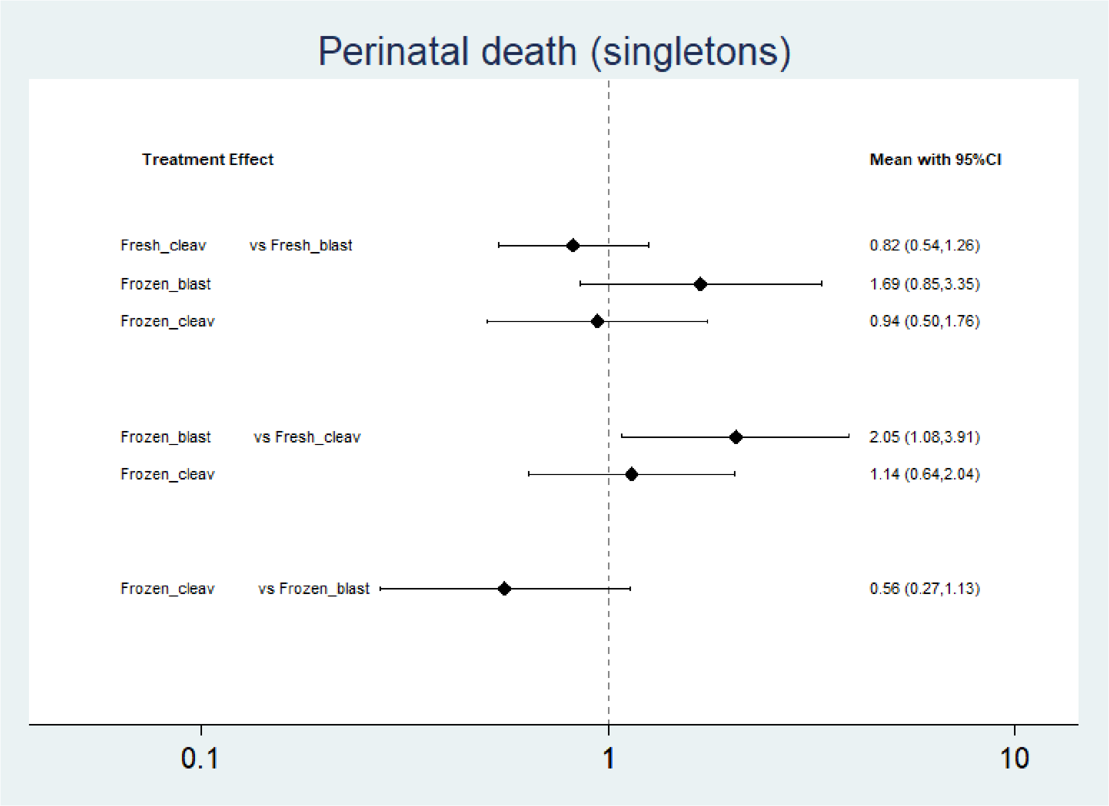

e. Gender of the baby (male)

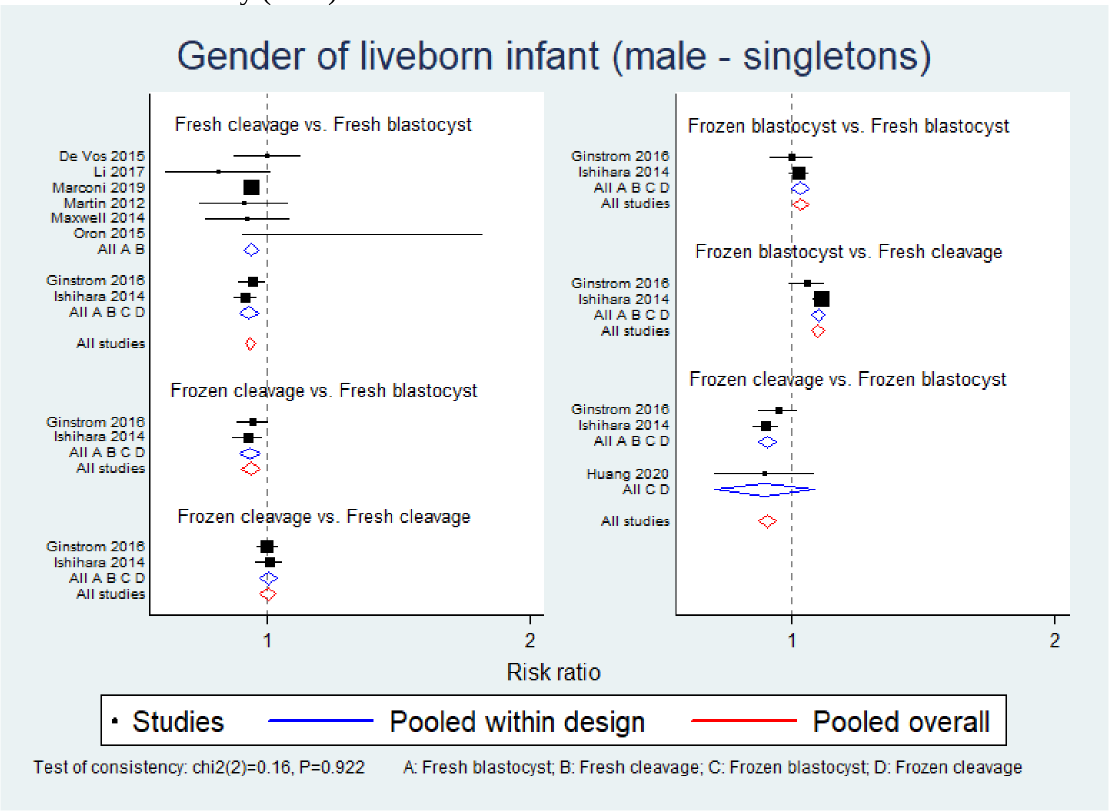

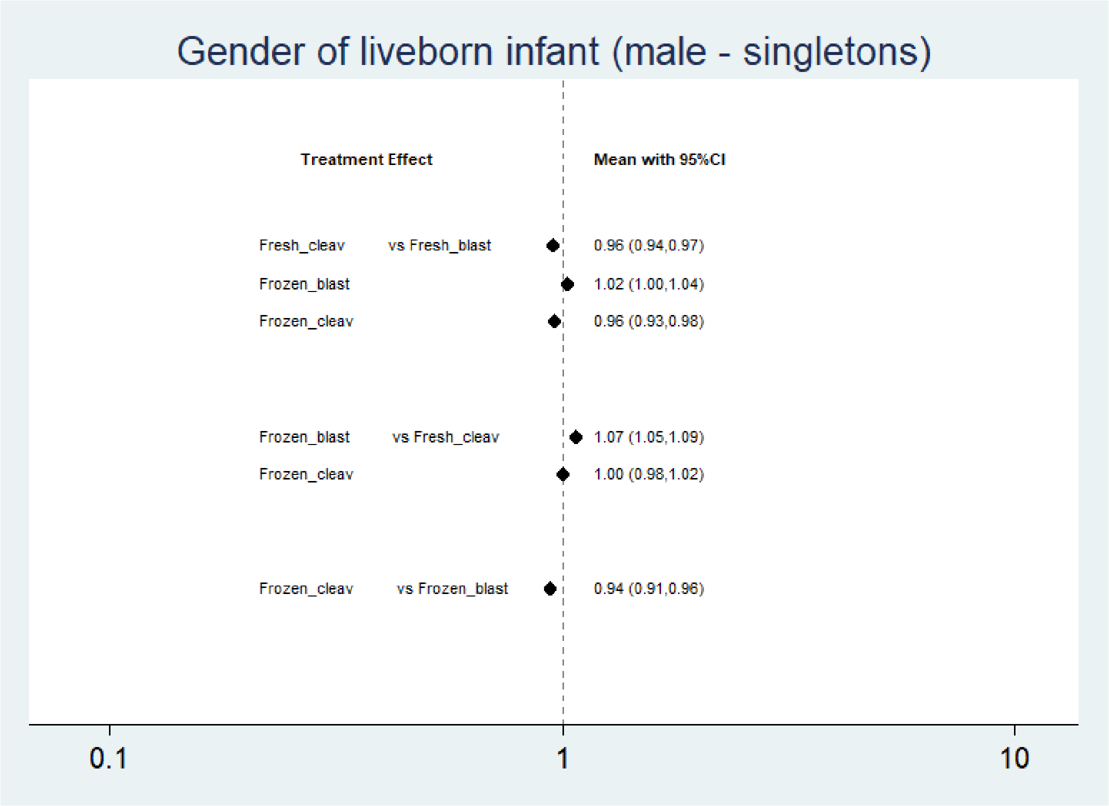

Gender of liveborn infant (male - singletons)
Treatment Effect
Mean with 95%CI
Fresh_cleav vs Fresh_blast
0.96 (0.94,0.97)
Frozen_blast
1.02 (1.00,1.04)
Frozen_cleav
0.96 (0.93,0.98)
Frozen_blast vs Fresh_cleav
1.07 (1.05,1.09)
Frozen_cleav
1.00 (0.98,1.02)
Frozen_cleav vs Frozen_blast
0.94 (0.91,0.96)
0.1
1
10

Supplementary figure 7 (Figure S7). Network meta-analyses for multiple pregnancies. Forest and interval plots for each outcome are presented. Only data on preterm delivery and low birth weight were sufficient to create connected networks. When considering an "Intervention 1" vs "Intervention 2" comparison (where interventions represent any 2 of the investigated modalities), a pooled effect on the right side of the x-axis would indicate that intervention 1 increases the risk for the examined outcome compared to intervention 2, while a pooled effect on the left side of the x-axis would indicate that intervention 1 decreases that risk compared to intervention 2. cleav, cleavage; blast, blastocyst.

a. Preterm delivery

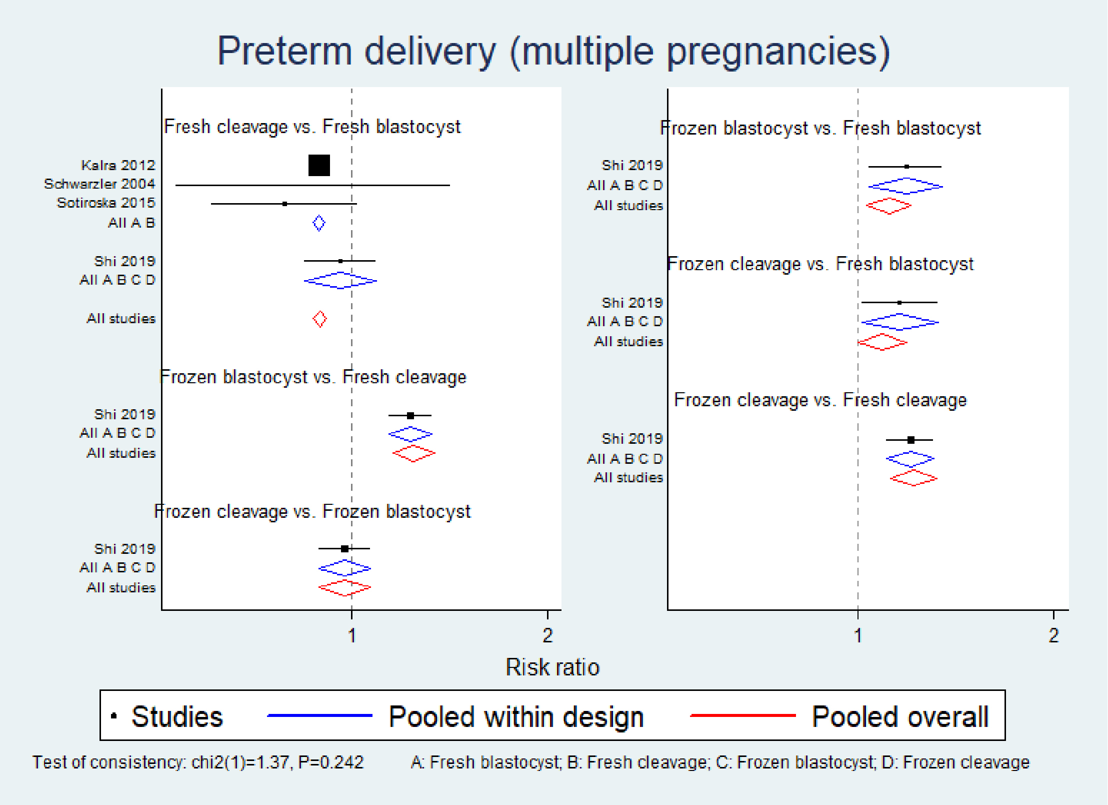

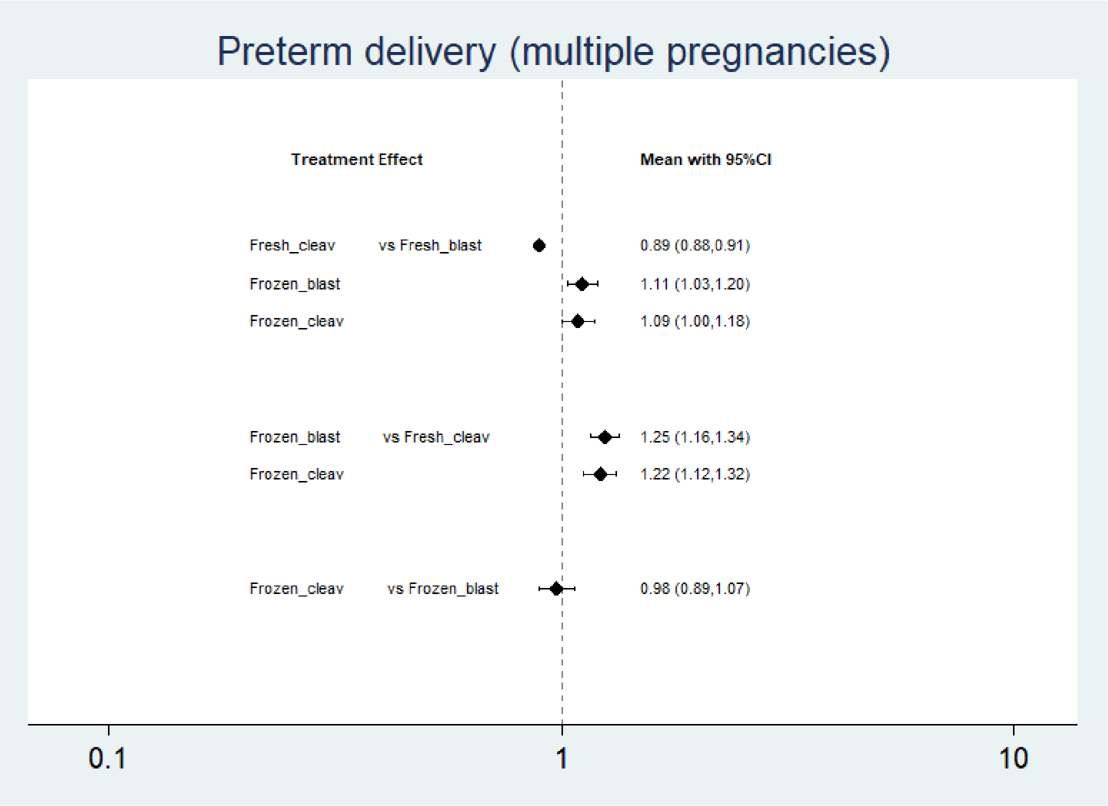

b. Low birth weight

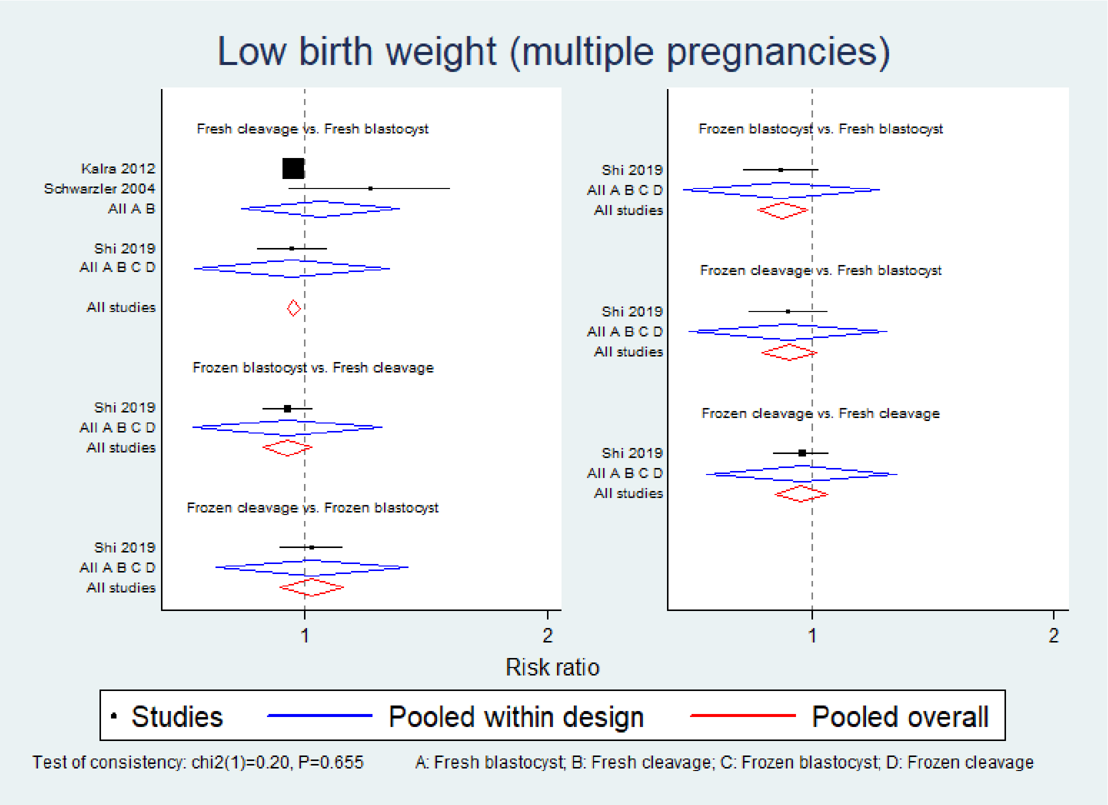

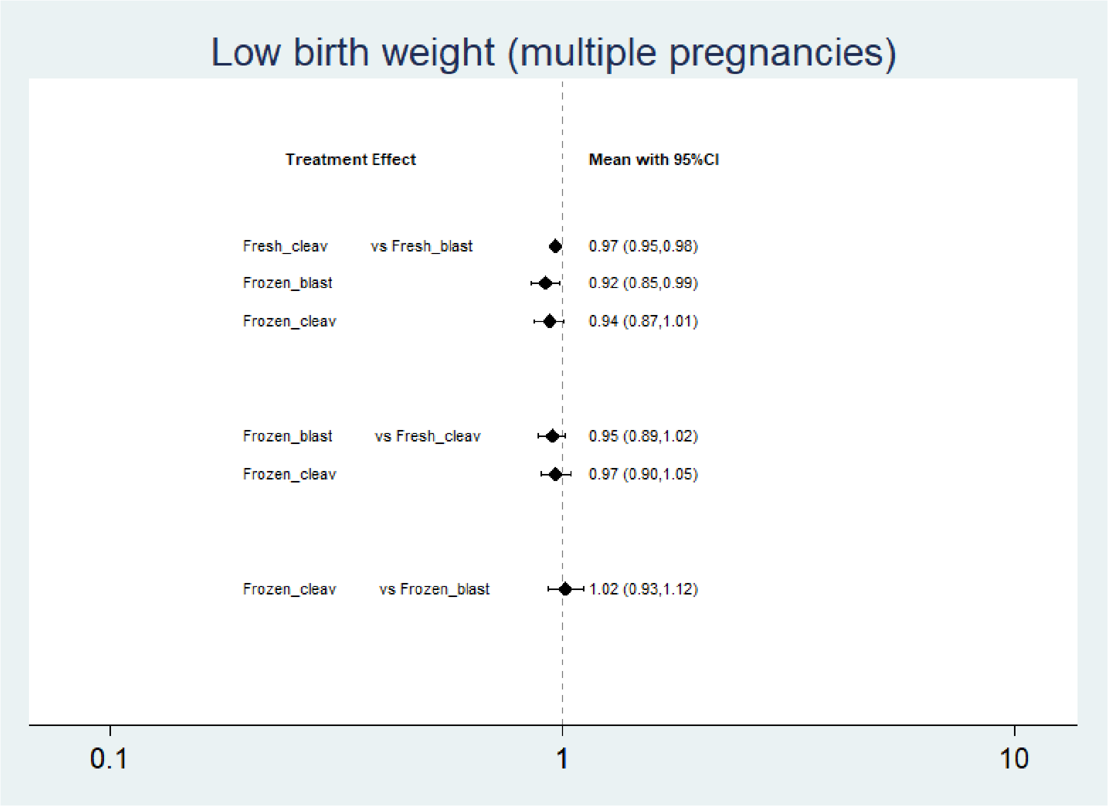

Low birth weight (multiple pregnancies)
Treatment Effect
Mean with 95%CI
Fresh_cleav vs Fresh_blast
0.97 (0.95,0.98)
Frozen_blast
0.92 (0.85,0.99)
Frozen_cleav
0.94 (0.87,1.01)
Frozen_blast vs Fresh_cleav
0.95 (0.89,1.02)
Frozen_cleav
0.97 (0.90,1.05)
Frozen_cleav vs Frozen_blast
1.02 (0.93,1.12)
0.1
1
10

Supplementary figure 8 (Figure S8). Network rankograms and cumulative ranking probabilities for identifying superiority of treatments: a. in reducing the risk for any congenital anomaly; fresh blastocyst demonstrated a 73.0% probability of being the best treatment and a 94.9% of being among the two best treatments, b. in reducing the risk for preterm delivery; fresh cleavage demonstrated a 45.7% probability of being the best treatment and a 75.4% of being among the best two treatments, c. in reducing the risk for low birth weight; frozen blastocyst demonstrated a 92.5% probability of being the best treatment and a 99.5% of being among the two best treatments, d. in reducing the risk for perinatal death; fresh cleavage demonstrated a 59.4% probability of being the best treatment and a 91.9% probability of being among the two best treatments, e. in reducing the probability of birth of male neonates; fresh cleavage demonstrated a 56.5% probability of being the best treatment and a 100% of being among the best two treatments.

a.

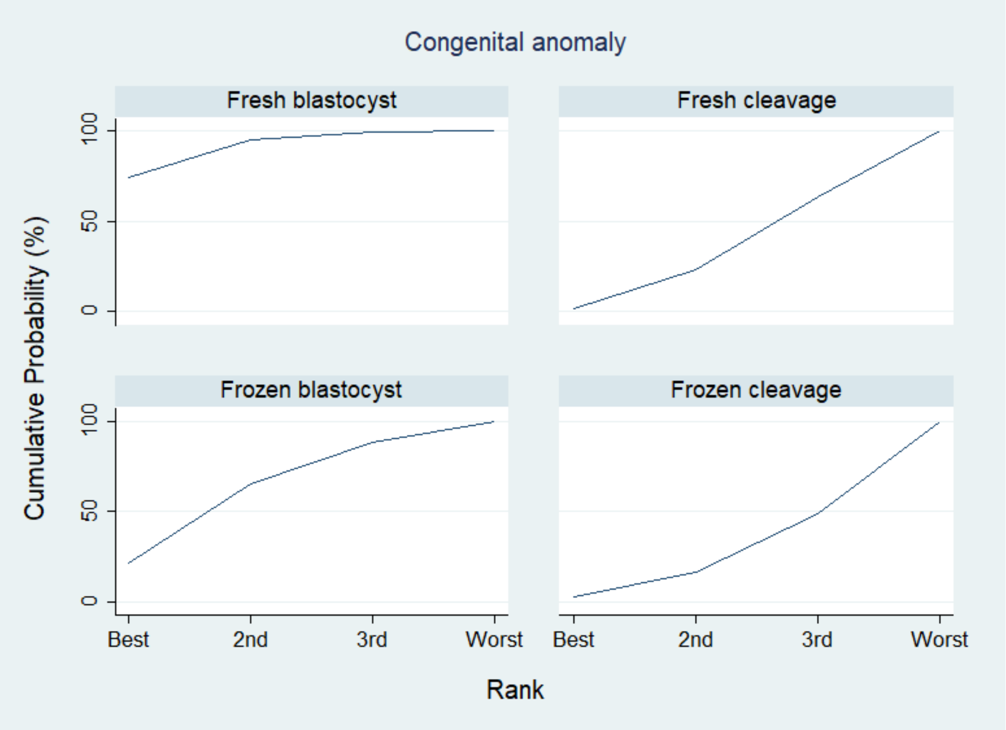

Rank	Treatment: Fresh blastocyst	Fresh cleavage	Frozen blastocyst	Frozen cleavage
Best	73.0	1.5	22.9	2.6
2nd	21.9	21.1	44.5	12.6
3rd	4.6	41.1	22.3	32.0
Worst	0.5	36.3	10.4	52.9

b.

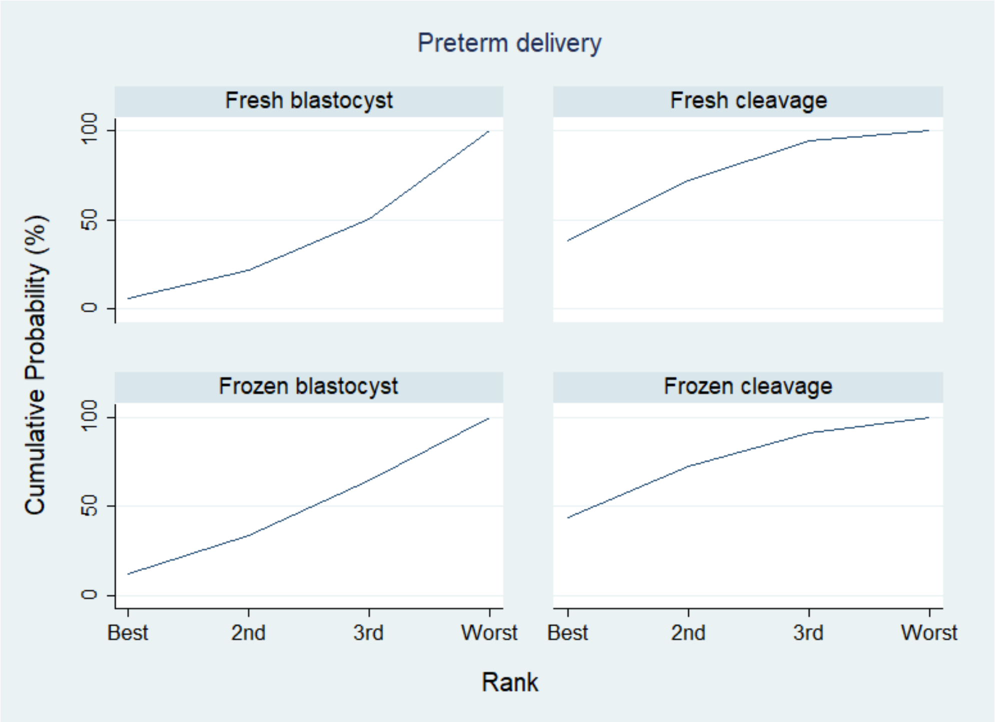

Rank	Fresh blastocyst	Fresh cleavage	Frozen blastocyst	Frozen cleavage
	Treatment			
Best	5.3	45.7	17.5	31.5
2nd	18.0	29.7	23.8	28.5
3rd	28.0	19.8	28.6	23.5
Worst	48.7	4.8	30.0	16.5

c.

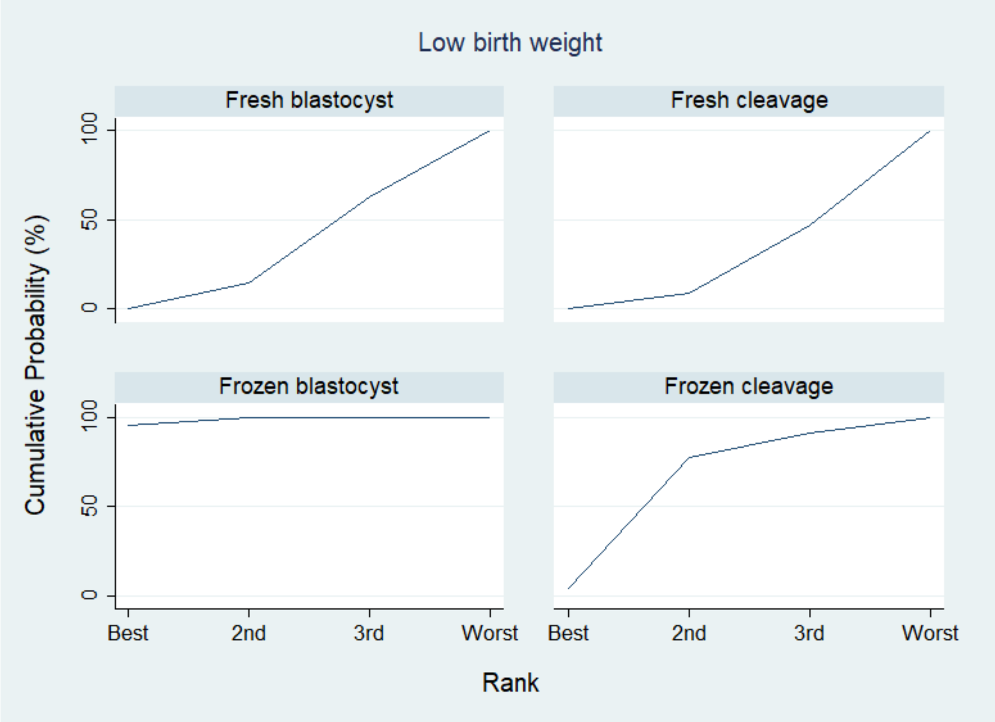

Rank	Treatment<br>Fresh blastocyst	Fresh cleavage	Frozen blastocyst	Frozen cleavage
Best	0.6	0.3	92.5	6.6
2nd	11.0	6.9	7.0	75.2
3rd	49.8	38.4	0.4	11.4
Worst	38.6	54.4	0.1	6.8

d.

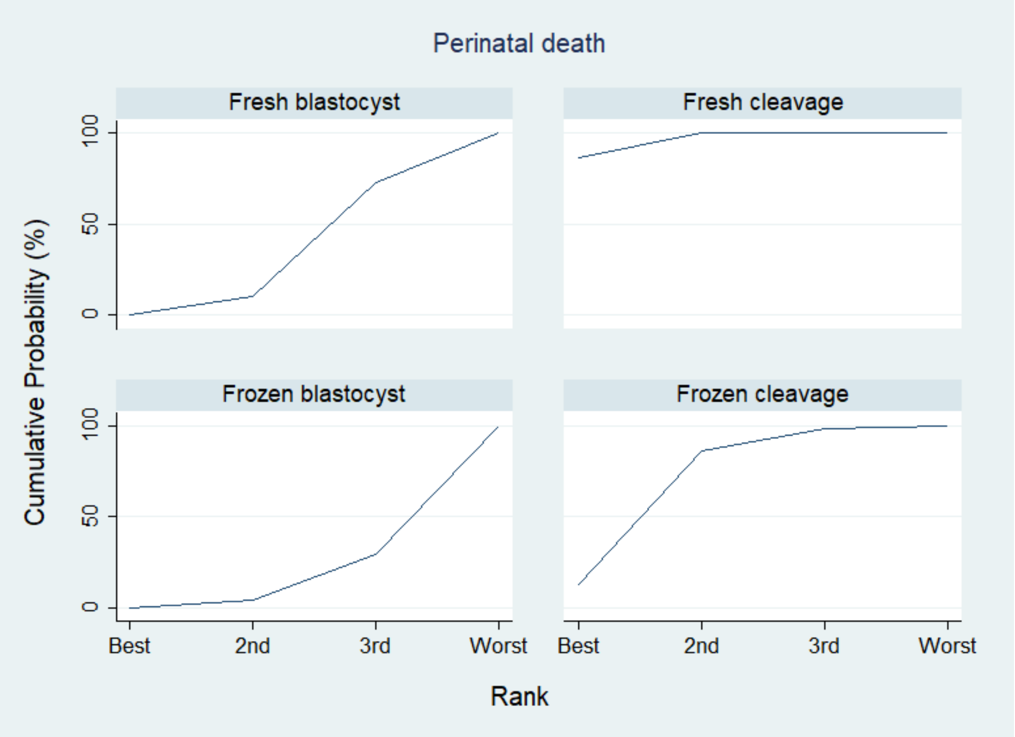

Rank	Treatment<br>Fresh blastocyst	Fresh cleavage	Frozen blastocyst	Frozen cleavage
Best	11.2	59.4	0.7	28.7
2nd	34.5	32.5	1.9	31.1
3rd	48.7	7.7	7.4	36.2
Worst	5.5	0.4	90.1	4.0

e.

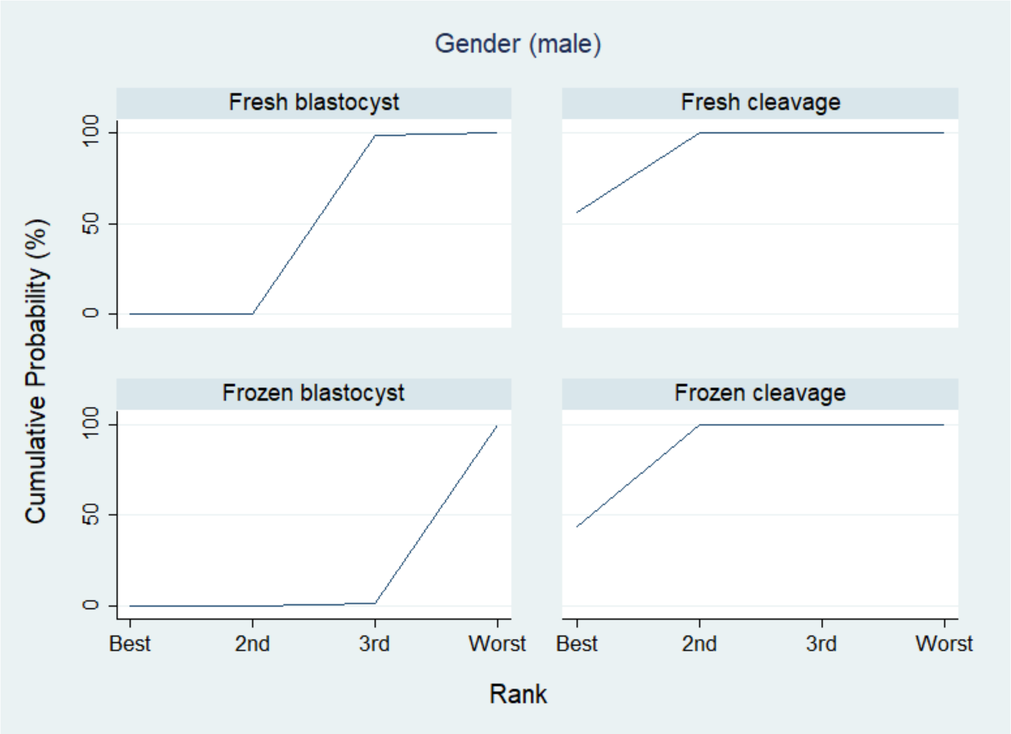

Rank	Treatment			
	Fresh blastocyst	Fresh cleavage	Frozen blastocyst	Frozen cleavage
Best	0.0	56.5	0.0	43.5
2nd	0.0	43.5	0.0	56.5
3rd	98.5	0.0	1.5	0.0
Worst	1.5	0.0	98.5	0.0

Supplementary figure 9 (Figure S9). Funnel plots of meta-analyses on any congenital anomaly (a), preterm delivery (gestational age at delivery < 37w) (b), c. low birth weight (< 2500 g) (c), perinatal death (d), gender (male) (e). Effect size (log risk ratio scale) is plotted against the corresponding standard error. Results of the Egger's regression test (p-value) for Funnel plot asymmetry are presented at the bottom left part of each figure.

a.

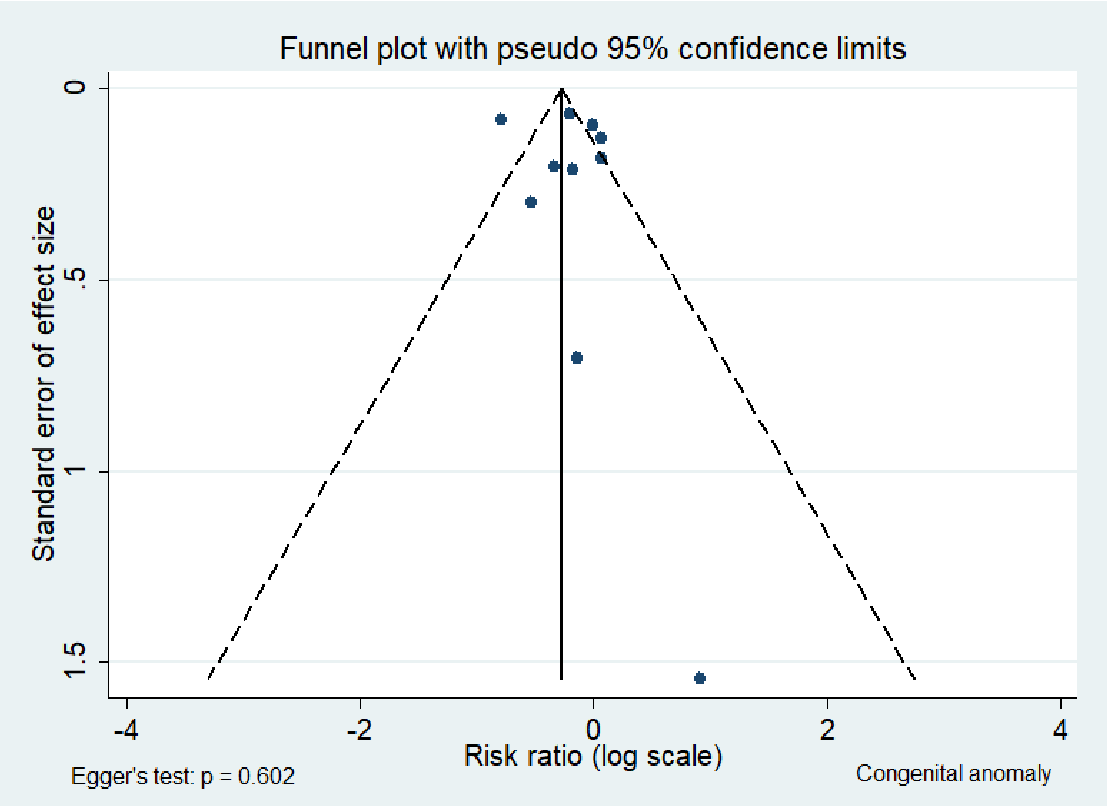

b.

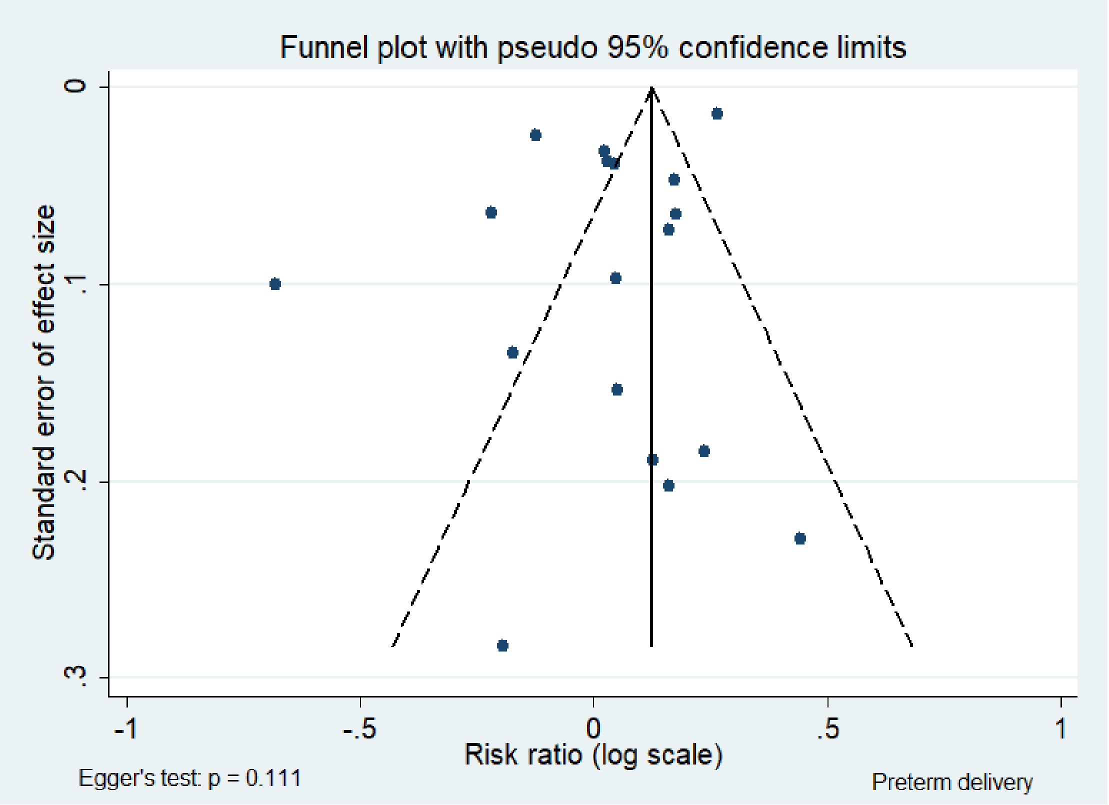

c.

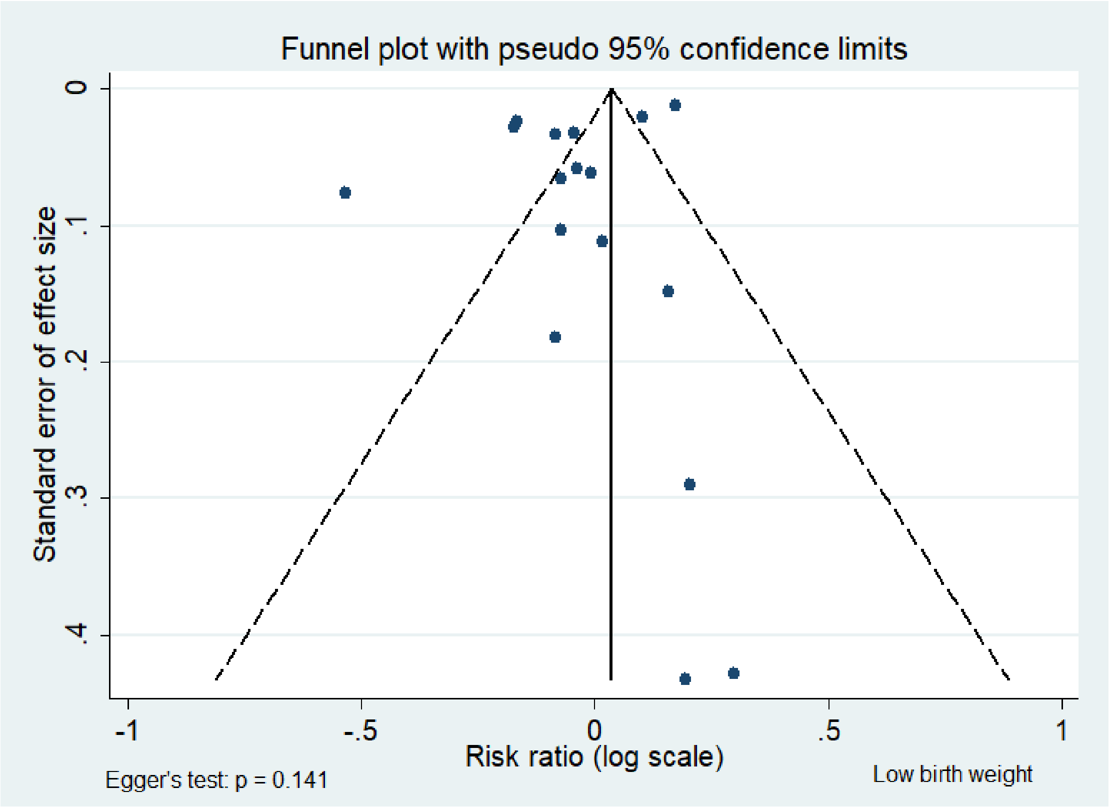
Funnel plot with pseudo 95% confidence limits
Standard error of effect size
0
.1
.2
.3
.4
-1
-.5
0
.5
1
Risk ratio (log scale)
Egger's test: p = 0.141
Low birth weight

d.

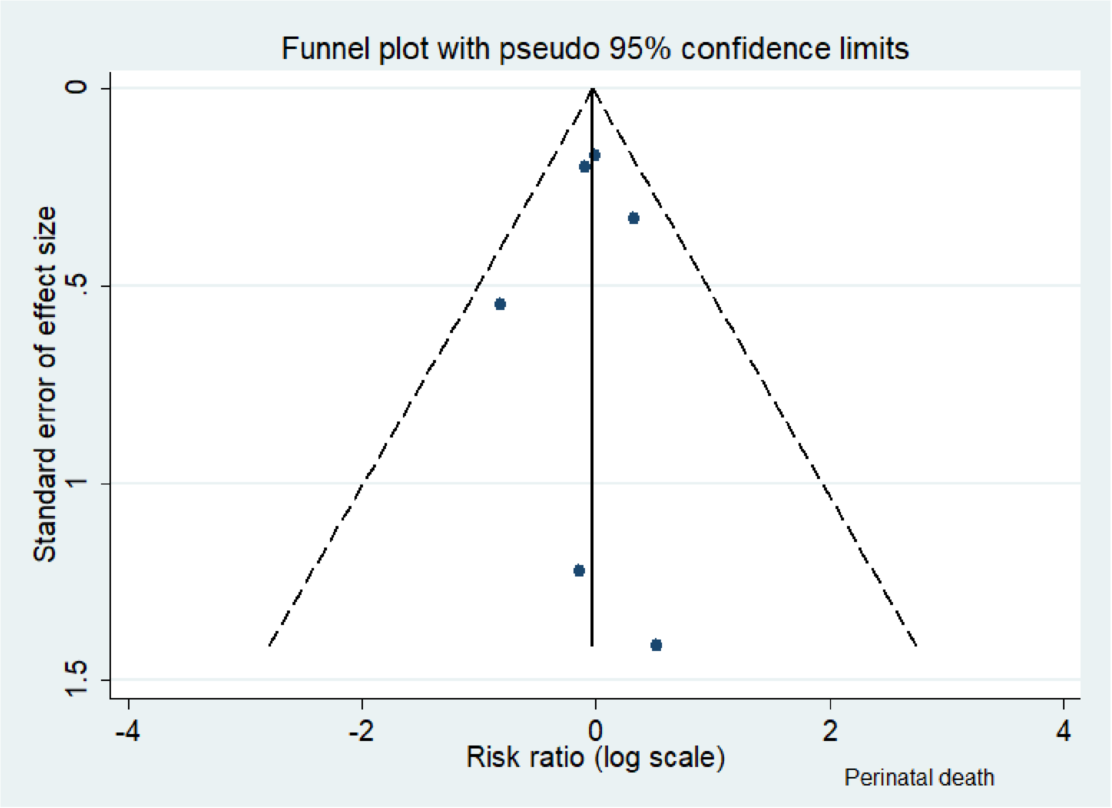
Funnel plot with pseudo 95% confidence limits
Standard error of effect size
0
.5
1
1.5
-4
-2
0
2
4
Risk ratio (log scale)
Perinatal death

e.

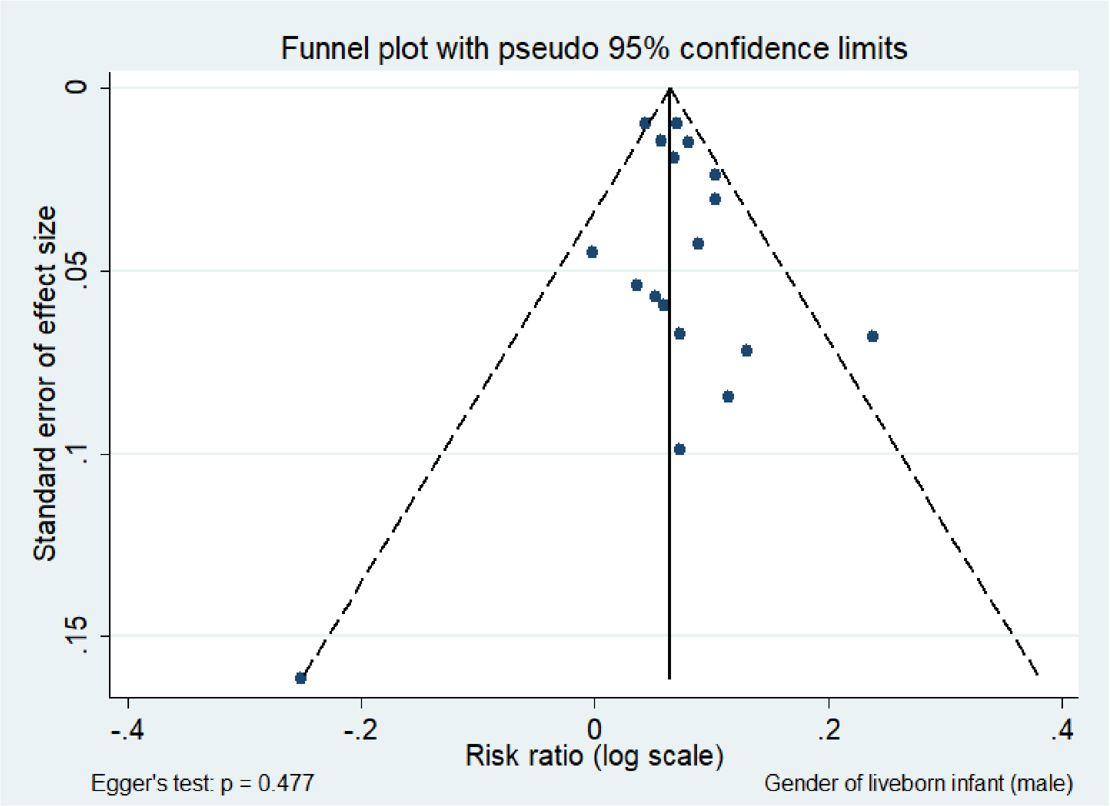
Funnel plot with pseudo 95% confidence limits
Standard error of effect size
0
.05
.1
.15
-.4
-.2
0
.2
.4
Risk ratio (log scale)
Egger's test: p = 0.477
Gender of liveborn infant (male)
